# Macrophage-derived chemokine CCL22 establishes local LN-mediated adaptive thermogenesis and energy expenditure

**DOI:** 10.1126/sciadv.adn5229

**Published:** 2024-06-26

**Authors:** Yexian Yuan, Ruoci Hu, Jooman Park, Shaolei Xiong, Zilai Wang, Yanyu Qian, Zuoxiao Shi, Ruifan Wu, Zhenbo Han, Sang-Ging Ong, Shuhao Lin, Krista A. Varady, Pingwen Xu, Daniel C. Berry, Gang Shu, Yuwei Jiang

**Affiliations:** ^1^ Department of Physiology and Biophysics, College of Medicine, University of Illinois at Chicago, Chicago, IL 60612, USA.; ^2^ Key Laboratory of Animal Genetics, Breeding and Reproduction of Shaanxi Province, College of Animal Science and Technology, Northwest A&F University, Yangling 712100, Shaanxi, China.; ^3^ Department of Pharmaceutical Sciences, University of Illinois at Chicago, Chicago, IL 60612, USA.; ^4^ Department of Microbiology and Immunology, University of Illinois at Chicago, Chicago, IL 60612, USA.; ^5^ Guangdong Laboratory of Lingnan Modern Agriculture, Guangdong Province Key Laboratory of Animal Nutritional Regulation and National Engineering Research Center for Breeding Swine Industry, College of Animal Science, South China Agricultural University, Guangzhou, 510642, China.; ^6^ Department of Pharmacology and Regenerative Medicine, College of Medicine, University of Illinois at Chicago, Chicago, IL 60612, USA.; ^7^ Division of Cardiology, Department of Medicine, University of Illinois at Chicago, Chicago, IL 60612, USA.; ^8^ Department of Kinesiology and Nutrition, University of Illinois at Chicago, Chicago, IL 60612, USA.; ^9^ Division of Endocrinology, Department of Medicine, University of Illinois at Chicago, Chicago, IL 60612, USA.; ^10^ Division of Nutritional Sciences, Cornell University, Ithaca, NY 14853, USA.

## Abstract

There is a regional preference around lymph nodes (LNs) for adipose beiging. Here, we show that local LN removal within inguinal white adipose tissue (iWAT) greatly impairs cold-induced beiging, and this impairment can be restored by injecting M2 macrophages or macrophage-derived C-C motif chemokine (CCL22) into iWAT. CCL22 injection into iWAT effectively promotes iWAT beiging, while blocking CCL22 with antibodies can prevent it. Mechanistically, the CCL22 receptor, C-C motif chemokine receptor 4 (CCR4), within eosinophils and its downstream focal adhesion kinase/p65/interleukin-4 signaling are essential for CCL22-mediated beige adipocyte formation. Moreover, CCL22 levels are inversely correlated with body weight and fat mass in mice and humans. Acute elevation of CCL22 levels effectively prevents diet-induced body weight and fat gain by enhancing adipose beiging. Together, our data identify the CCL22-CCR4 axis as an essential mediator for LN-controlled adaptive thermogenesis and highlight its potential to combat obesity and its associated complications.

## Teaser

Macrophage-derived chemokine CCL22 and its receptor CCR4 play a pivotal role in facilitating thermogenesis in response to cold.

## INTRODUCTION

Brown and beige adipocytes consume large amounts of glucose and lipids, converting the resulting chemical energy into heat, thus offering therapeutic potential for patients with obesity and diabetes (*1*–*4*). Compared to classical brown adipocytes, which are constitutively present, beige adipocytes are minimally present at room temperature (RT) and require de novo biogenesis from adipocyte progenitor cells (APCs) or transformation of white adipocytes within white adipose tissue (WAT) in response to cold stimuli (*5*–*14*). Despite extensive research into the molecular pathways underlying beige adipocyte biogenesis (*15*–*17*), there remains a limited understanding of how APCs receive and respond to signals from their local microenvironment. This knowledge gap is important because the capacity of APCs to undergo beiging in WAT decreases with aging and obesity, which is associated with a loss of their ability to expend energy (*18*–*26*).

Recent studies have demonstrated that beige adipocytes can only be activated in specific regions in both rodents and humans. For example, cold stimuli can substantially recruit beige adipocytes within inguinal WAT (iWAT) but not within visceral WAT. Moreover, within iWAT, the area surrounding the local lymph nodes (LNs) in the tissue’s center appears to be the most effective region for beige adipocyte activation (*27*–*30*). In addition, beige adipocytes near the LN area are readily detectable during the postnatal period, even at RT (*31*, *32*). Nevertheless, the role of LNs in iWAT beiging has not been explored until recently. A very recent publication reveals the critical role of LNs in iWAT beiging (*33*). They found that cold exposure enhances sympathetic nervous system (SNS) activity in LNs, promoting the release of interleukin-33 (IL-33) from fibroblastic reticular cells to the surrounding iWAT, thereby activating type 2 innate lymphoid cells to induce beiging (*33*). However, the mechanisms by which the local immune microenvironment and APCs work together to regulate the beiging and adaptive thermogenic response of iWAT to cold exposure are still not fully understood.

Emerging evidence suggests that immune cells, particularly adipose tissue macrophages, regulate adaptive thermogenesis and energy expenditure (*34*–*37*). Macrophages are highly versatile cells that display a remarkable degree of plasticity. Within iWAT, they are primarily categorized into two main types: M1-like macrophages, which are classically activated and pro-inflammatory, and M2-like macrophages, which are alternatively activated and anti-inflammatory. In obesity, adipose tissue experiences a shift from anti-inflammatory M2 to pro-inflammatory M1-like macrophages, contributing to insulin resistance. In contrast, M2 macrophages are prevalent in lean adipose tissue and produce anti-inflammatory cytokines such as transforming growth factor–β and IL-10 (*38*, *39*). To date, accumulated evidence shows a strong association between the expansion of M2 macrophages and beige adipocyte activation in response to cold stimuli (*34*, *36*, *40*–*44*). However, the role of the macrophages in iWAT beiging remains controversial. For instance, other macrophage subsets in adipose tissue can hinder adaptive thermogenesis and elicit the pathogenesis of obesity (*45*, *46*). It is therefore important to understand the molecular processes by which M2 macrophages expand upon exposure to cold and how this expansion affects beige adipocyte biogenesis.

One mechanism for regulating macrophage expansion and activity in iWAT involves the secretion of chemokines and their receptors. This includes C-C motif chemokine 22 (CCL22), also known as macrophage-derived chemokine (MDC). CCL22 influences target cells by binding to the CC chemokine receptor 4 (CCR4) (*47*). CCR4 is primarily expressed in eosinophils and several T cell subsets, including T helper 2 (T_H_2) cells (*48*). Evidence suggests that CCL22 and its receptor CCR4 may be involved in the beige adipocyte biogenesis and energy regulation in iWAT in response to cold temperatures (*49*). For example, cold exposure increases CCL22 expression in iWAT, while a high-fat diet (HFD) decreases CCL22 expression (*49*). Despite these observations, the precise roles of CCL22 and CCR4 within adipose immune cells in activating thermogenic beige adipose tissue have yet to be determined.

Here, we report the crucial role of local LNs, MDC CCL22, and its receptor CCR4 in enabling perivascular APC beiging. Specifically, inguinal LN removal hinders cold-induced beiging, which can be restored by administering M2 macrophages or CCL22 into iWAT. Furthermore, CCL22 levels in iWAT and serum progressively increase in response to cold stimuli. In line with its expression, CCL22 injection into iWAT effectively promotes iWAT beiging, while blocking CCL22 with antibodies can prevent it. Mechanistically, the CCL22 receptor CCR4 within eosinophils and its downstream FAK/p65 signaling pathway are essential for CCL22-mediated beige adipocyte formation. Last, both mouse and human serum CCL22 levels show an inverse correlation with body weight and fat mass. In humans, weight loss interventions increase serum CCL22 levels. In mice, increasing CCL22 levels prevents diet-induced obesity (DIO) and increases energy expenditure. Thus, CCL22, through its interaction with its receptor CCR4, establishes the local microenvironment to regulate adipose beiging and energy homeostasis, offering therapeutic potential for preventing obesity and related metabolic disorders.

## RESULTS

### Inguinal lymph node removal compromises cold-induced accumulation of M2 macrophages

**Fig. 1.**
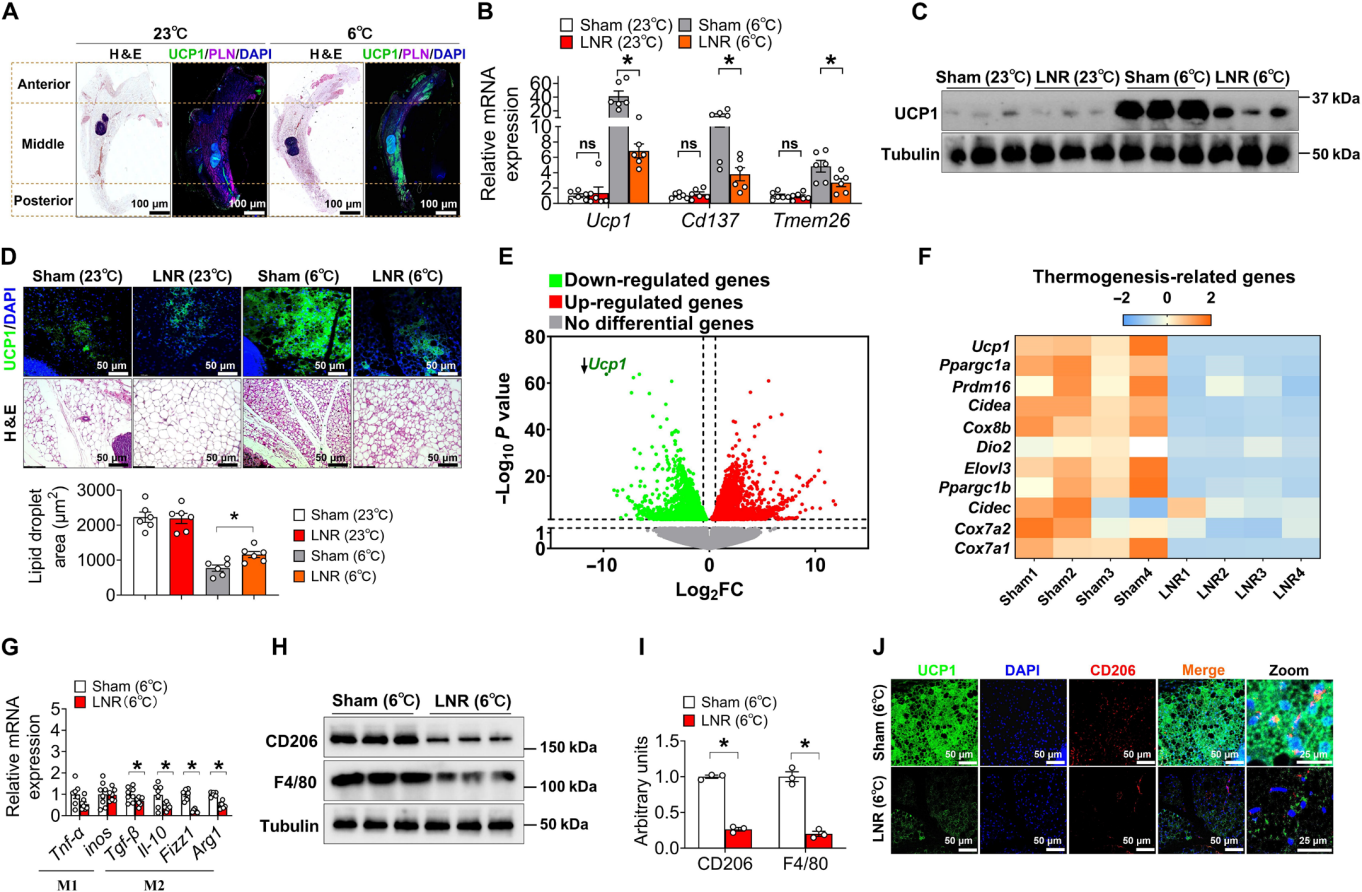
Lymph node removal impairs cold-induced iWAT beiging and a lack of M2 macrophage accumulation. (**A**) H&E staining and UCP1 staining of inguinal adipose tissue (iWAT). Ten-week-old male C57BL/6 mice were housed at RT (23°C) or cold exposure (6°C) for 7 days with a chow diet. Scale bars, 100 μm. (**B** and **C**) mRNA expression (B) of *Ucp1*, *Cd137*, and *Tmem26*, and immunoblots (C) of UCP1 in the middle part of iWAT (*n* = 3 to 6 per group). (**D**) Immunofluorescence of UCP1 and H&E staining and quantification of lipid droplet size in the middle of iWAT. Scale bars, 50 μm. (**E**) A volcano plot of genes. Ten-week-old male C57BL/6 mice were divided into two groups with sham or bilateral LN (inlaid in iWAT) removal (LNR) stimulated by cold exposure (6°C) for 7 days with a chow diet (*n* = 4 per group). (**F**) Heatmap of a list of thermogenic gene expression. (**G**) M1 and M2 macrophage marker gene expression in iWAT from 10-week-old C57BL/6 male mice exposed to 6°C for 7 days with chow feeding (*n* = 7 to 8 per group). (**H** and **I**) Immunoblots (H) and quantification (I) of CD206 and F4/80 in iWAT from 10-week-old C57BL/6 male mice exposed to 6°C for 7 days with chow feeding. (**J**) Immunofluorescence of UCP1 and CD206 in iWAT from 10-week-old C57BL/6 male mice exposed to 6°C for 7 days with chow feeding. Scale bars, 50 μm. Data information: Results are presented as means ± SEM. (B, G, and I) *P ≤ 0.05 by nonpaired Student’s *t* test. DAPI, 4′,6-diamidino-2-phenylindole. PLN, perilipin 1.

Cold-induced beige adipocytes are primarily formed around the inguinal LNs in the middle of iWAT, as revealed by histological examination using hematoxylin and eosin (H&E) and immunofluorescence (IF) staining of uncoupling protein 1 (UCP1) (Fig. 1A). To confirm the importance of local LNs in beige adipocyte formation, we performed inguinal sham or lymphadenectomy/LN removal (LNR) surgery on 10-week-old C57BL/6 male mice at RT (23°C). After 10 days of recovery, all mice were subjected to a cold challenge of 6°C for 7 days to induce iWAT beiging (fig. S1A). Consistent with a recent finding (*33*), the mRNA levels of thermogenic genes (*Ucp1*, *Cd137*, and *Tmem26*) by quantitative real-time polymerase chain reaction (qPCR) analysis were reduced in the middle parts of iWAT in LNR mice (Fig. 1B). Accordingly, comparable loss of beiging responses was revealed in the LNR group by H&E, UCP1 IF staining, quantification of lipid droplet size, and Western blotting (Fig. 1, C and D, and fig. S1B).

Next, to pinpoint the molecular alterations in iWAT beyond the LN region following LNR, we extracted the RNAs from the iWAT of the sham and LNR groups for RNA sequencing (RNA-seq). Of note, the LNs from the sham group were also physically removed from iWAT when the samples were prepared. A total of 8005 genes were identified to be changed by more than >1.3 fold, of which 4141 were reduced in the LNR cold exposure group. Among the list of the reduced genes, we identified *Ucp1* as a top target gene (Fig. 1E), and thermogenic genes were decreased evidently (Fig. 1F).

Since immune function is well known to be regulated by LN, we performed cluster and Gene Ontology (GO) analyses related to immune function between sham and LNR groups. The genes of both innate and adaptive immune responses were markedly altered by LNR (fig. S1C). Among the various cellular components of immune responses, macrophage activation was differentially regulated, but not the other components, including neutrophil, mast cell, natural killer T cell, or dendritic cell (DC) between sham and LNR groups (fig. S1D). Of note, while eosinophil migration exhibited a trend, it was not statistically substantial. These results suggest that sham and LNR groups may differ primarily in macrophage activation and function. Consistent with the GO analyses, LNR mice also showed decreased mRNA expression of *Tgf-*β, *Il-10*, *Arg1*, and *Fizz1*, mainly produced by M2 macrophages in the iWAT (Fig. 1G). In contrast, M1-like macrophage markers (*Tnf-*α and *iNOS*) remained unchanged in iWAT (Fig. 1G). Together, these data led us to speculate that M2 macrophages are involved in the regulatory effects of the LNs on cold-induced adaptive thermogenesis.

M2 macrophages, defined as F4/80^hi^CD45^+^CD64^+^CD206^+^ cells, were shown to be recruited during the cold challenge (*41*). This was confirmed by our fluorescence-activated cell sorting (FACS) data using stromal vascular fraction (SVF) cells minus LN isolated from iWAT of 10-week-old C57BL/6 male mice. Specifically, cold exposure for 7 days increased the frequency of M2 macrophages in iWAT by approximately twofold relative to the RT controls (fig. S1, E to G). The cold-induced increase in M2 macrophages was largely abolished in the LNR group, whereas LNR at RT did not affect the ratio of M2 macrophages in iWAT (fig. S1, E to G). In addition, consistent with the reduction of M2 macrophages, we also observed reduced protein levels of CD206 and F4/80 based on Western blotting and IF staining in iWAT tissues, isolated SVF cells, and bone marrow–derived macrophages (BMDMs) from the LNR mice (Fig. 1, H to J, and fig. S1H). To further support the role of local LNs in beiging activity, we used a Seahorse bioanalyzer to assess the oxygen consumption rate (OCR) of beige adipocytes. Our observations revealed that beige adipocytes originating from LNR-SVF cells exhibited a decrease in OCR when compared to those derived from sham-SVF cells (fig. S1, I and J). Together, these data indicate that local LNs may serve as a hub for recruiting M2 macrophages into iWAT during the cold challenge, and the loss of this immune recruitment may lead to the failure of iWAT beiging in response to cold exposure.

### M2 macrophage injection restores cold-induced beiging in LNR mice

**Fig. 2.**
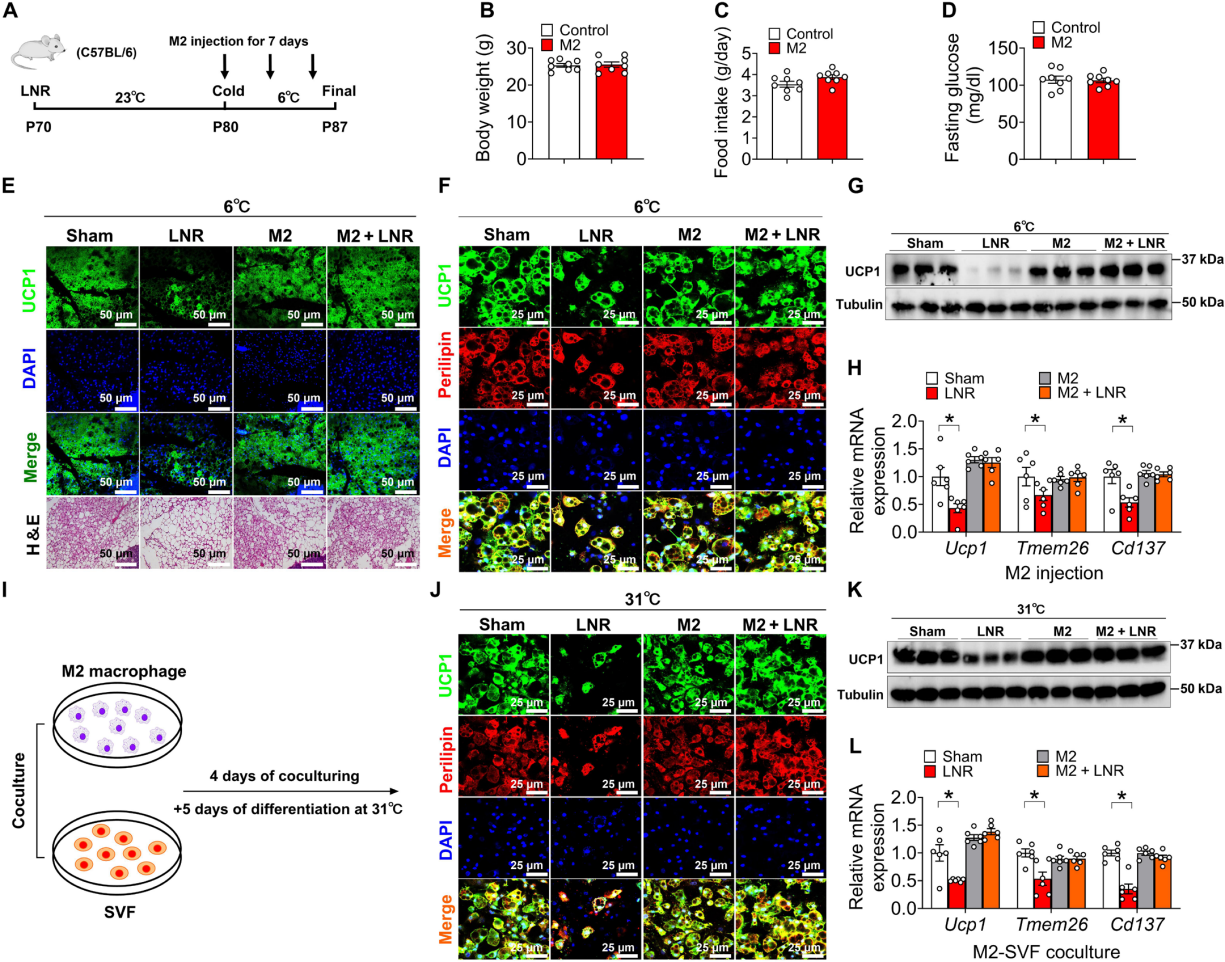
M2 macrophages restore LNR-impaired cold-induced iWAT beiging. (**A**) Schematic representation of M2 macrophage injection into iWAT. Ten-week-old C57BL/6 male mice were exposed to 6°C, and M2 macrophages were injected into bilateral iWAT for 7 days with chow feeding (*n* = 8 per group). (**B** to **D**) Body weight (B), food intake (C), fasting glucose (D) (*n* = 8 per group). (**E** to **H**) Immunofluorescence of UCP1 in iWAT (E), IF of UCP1 in beige adipocytes (F), immunoblots of UCP1 (G), and mRNA expression of *Ucp1*, *Cd137*, and *Tmem26* (H) in iWAT (*n* = 3 to 6 per group). Scale bars, (E) 50 and (F) 25 μm. (**I**) Schematic representation. M2 macrophages and SVF cells were cocultured for 4 days, and beige adipocytes (with M2 macrophage cocultured) were then induced for 5 days at 31°C. SVF cells were obtained from 10-week-old C57BL/6 male mice that received sham or LNR. M2 macrophages were obtained from 10-week-old C57BL/6 male mouse bone marrow. (**J** to **L**) Immunofluorescence (J), immunoblots (K) of UCP1, and mRNA expression (L) of *Ucp1*, *Cd137*, and *Tmem26* in beige adipocytes (*n* = 3 to 6 per group). Scale bars, 25 μm. Data information: Results are presented as means ± SEM. [(H) and (L)] *P ≤ 0.05 by nonpaired Student’s *t* test.

We next investigated whether the iWAT-specific M2 macrophage replenishment could rescue the LNR-induced failure in cold-induced beiging. Specifically, the iWAT LNs of 10-week-old C57BL/6 male mice were first surgically removed, and the mice were exposed to cold temperature (6°C) with bidaily local injection of either vehicle or M2 macrophages into iWAT for a total of three times during the 7-day cold exposure (Fig. 2A). We found that M2 macrophage injection did not affect body weight, body composition, food intake, or fasting glucose (Fig. 2, B to D, and fig. S2, B and C). However, injection of M2 macrophages increased body core temperature (fig. S2A) and showed restored beige adipocyte formation in LNR iWAT upon cold exposure, as assessed by UCP1 IF staining, Western blotting, and mRNA expression of thermogenic genes (Fig. 2, E to H). Injection of M2 macrophages did not further promote cold-induced beiging in the sham group (Fig. 2, E to H).

To provide evidence validating that the injection of exogenous M2 macrophages is successful, we used red fluorescent protein (RFP)–labeled M2 macrophages for iWAT injection and evaluated their survival at one and two days after injection. Our flow cytometry analysis indicated that a notable number of these RFP-M2 macrophages remain evident on days 1 and 2 following the injection (fig. S2D), indicating that the exogenously injected M2 macrophages are still alive within iWAT.

To further examine the role of M2 macrophages in LN-mediated iWAT beiging, we cocultured SVF cells with M2 macrophages at cold (31°C) instead of warm (37°C) temperature (Fig. 2I), a previously established cooling procedure to robustly induce SVF beiging in vitro (*50*). First, we monitored the cell viability of M2 macrophages incubated at 31° and 37°C. Our data indicated that there is no substantial difference in cell viability on days 1, 3, and 7 between those two temperatures, including M2 macrophages, eosinophils, and DCs (fig. S3). Consistent with our in vivo data, coculturing SVF cells with M2 macrophages restored SVF beiging isolated from the LNR mice, as assessed by UCP1 IF staining, Western blotting, and expression of thermogenic genes (Fig. 2, J to L). These results demonstrate that direct injection of M2 macrophages into iWAT can bypass the requirement of local LNs in driving the downstream events for beiging. Thus, our data support the notion that upon cold exposure, M2 macrophages are expanded within iWAT by the LNs and hence promote iWAT beiging.

### Cold-induced CCL22 predominantly originates from M2 macrophages

**Fig. 3.**
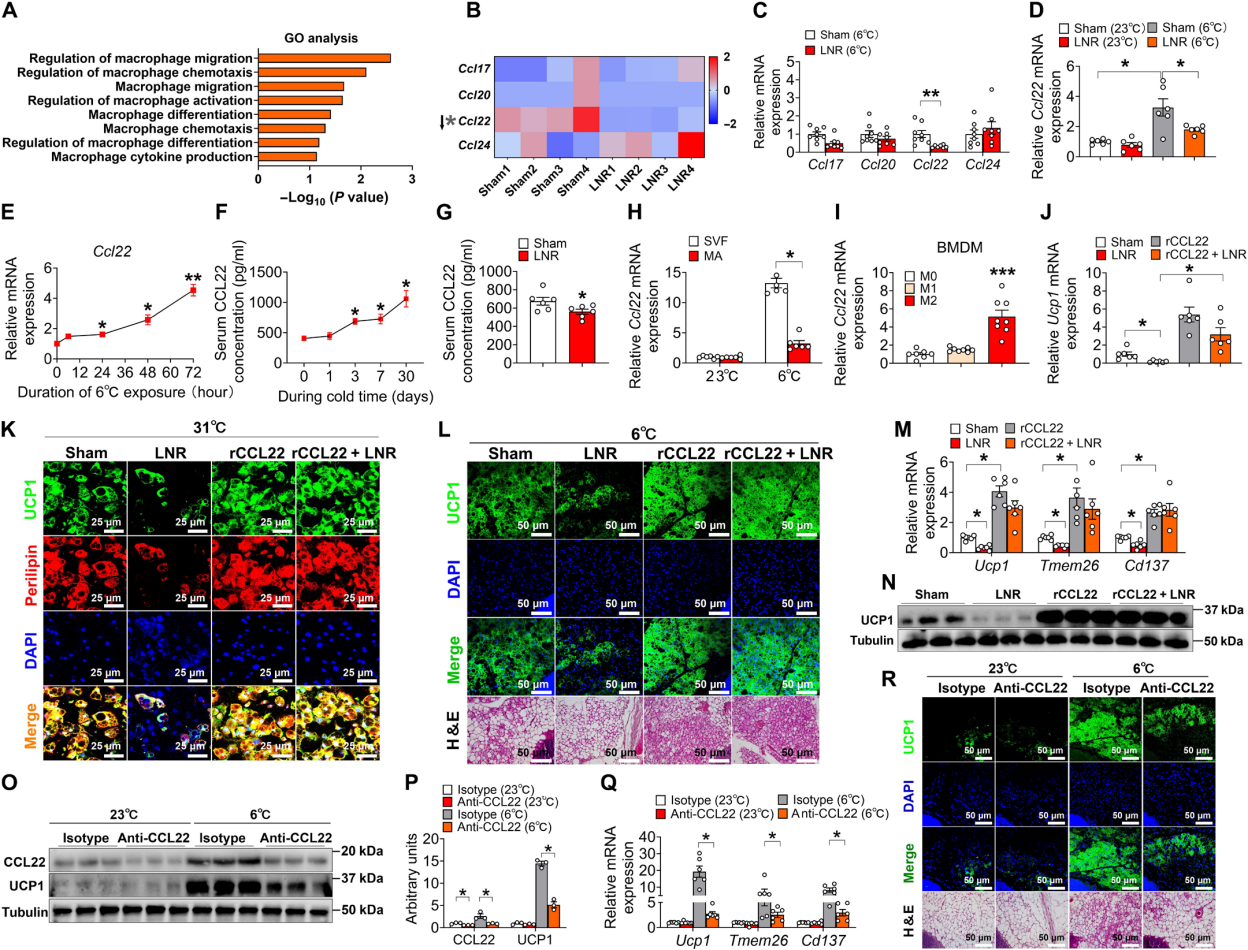
Macrophage-induced CCL22 facilitates LN-mediated cold-induced iWAT beiging. (**A**) GO analysis of macrophage response to LNR. (**B**) Heatmaps of macrophage-secreted chemokines in the iWAT from mice receiving sham or LNR at 6°C for 7 days (*n* = 4 per group). Red and blue represent the fold increase and decrease in a gene, respectively (see color scale). (**C**) mRNA expression of macrophage-secreted chemokines in iWAT from mice described in (B) (*n* = 8 per group). (**D**) *Ccl22* mRNA expression in iWAT from mice described in (B) at 23° or 6°C for 7 days (*n* = 6 per group). (**E**) *Ccl22* mRNA expression in iWAT from mice described in (B) exposed to 6°C for different hours (*n* = 6). (**F**) Serum CCL22 levels from mice described in (B) exposed to 6°C for different days (*n* = 6). (**G**) Serum CCL22 levels from mice described in (B) (*n* = 6 per group). (**H**) *Ccl22* mRNA expression in iWAT SVF cells or mature adipocytes (MAs). (**I**) *Ccl22* mRNA expression in M0, M1, and M2 macrophages derived from 10-week-old C57BL/6 bone marrows (*n* = 8 per group). (**J** and **K**) mRNA expression (J) and IF (K) of UCP1 in beige adipocytes (*n* = 6 per group). Scale bars, 25 μm. (**L** to **N**) Immunofluorescence of UCP1 (L), mRNA expression of thermogenic genes (M), and immunoblots of UCP1 (N) in iWAT (*n* = 3 to 6 per group). Scale bars, 50 μm. (**O** to **R**) Immunoblots and quantification of UCP1 [(O) and (P)], mRNA expression (Q) of thermogenic genes, and IF of UCP1 and H&E staining (R) in iWAT (*n* = 3 to 6 per group). Scale bars, 50 μm. Data information: Results are presented as means ± SEM. [(C), (D), (G) to (J), (M), (P), and (Q)] *P ≤ 0.05, **P < 0.01, and ***P < 0.001 by nonpaired Student’s *t* test. [(E) and (F)] *P ≤ 0.05 and **P < 0.01 nonpaired Student’s *t* test compared with before cold stimulation.

To further dissect the importance and the molecular consequences of macrophage expansion in iWAT beiging, we performed the GO analysis related to macrophage genes. We observed that genes that regulate macrophage migration and chemotaxis were ranked at the top (Fig. 3A). Therefore, we examined the expression levels of genes relating to macrophage-derived cytokines, chemokines, and their regulators from our RNA-seq data. Among them, the chemokine CCL22 was the most notably down-regulated molecule in the iWAT from LNR mice compared to that of the sham group (Fig. 3B), which was further confirmed by qPCR analysis (Fig. 3C). Moreover, we found that cold temperature markedly increased *Ccl22* mRNA levels in iWAT (Fig. 3, D and E). Corresponding to an increase in the level of *Ccl22* mRNA, the secretion of CCL22 protein was also substantially increased throughout cold exposure (Fig. 3F). Consistent with the RNA-seq data, the cold-induced mRNA and serum CCL22 elevation were abolished in the LNR group (Fig. 3, D to G). Further cell fractional analysis within iWAT showed that cold-induced CCL22 expression occurred predominantly in the SVF cells but not in mature adipocytes (MAs; Fig. 3H). To examine this further within macrophage subsets, analysis of the successfully differentiated M1 versus M2 macrophages from BMDMs indicated that CCL22 is more highly expressed in M2 compared to M1 macrophages (Fig. 3I). Together, our data show a selective induction of CCL22 expression in M2 macrophages upon cold exposure, suggesting that local CCL22 induction in iWAT may play a critical role in regulating LN-mediated iWAT beiging.

While CCL22 is mainly secreted by M2 macrophages (*51*), CCL22 is also secreted by DCs according to a previous study (*52*). Thus, we checked the alterations in DC markers and the expression of CCL22 in DCs. Our flow cytometry data showed that the DC markers (CD11c, I-A/I-E, and CD103) did not change after LNR (fig. S4, A and B). In addition, CCL22 levels in DCs remained unaltered upon LNR, as assessed by both a CCL22/MDC enzyme-linked immunosorbent assay (ELISA) and Western blot analysis (fig. S4, C to E). Together with our macrophage rescue data, our results strongly suggest that M2 macrophage–derived CCL22, but not that from DCs, mediates the LN-controlled iWAT beiging.

To further narrow down cold-induced CCL22 production predominantly originating from M2 macrophages, we first examined CCL22 expression in cells known to produce CCL22, including M2 macrophages, DCs, and eosinophils at both RT and after cold exposure. Our findings indicate that cold exposure triggers up-regulation of CCL22 expression in both DCs and M2 macrophages, with the most pronounced increase observed in M2 macrophages (fig. S5A). Next, to probe the role of CCL22 induction in M2 macrophages, we treated bone marrow–derived M2 macrophages with CCL22 small interfering RNA (siRNA), followed by coculture experiments. Our findings suggest that silencing CCL22 in these M2 macrophages abolishes their capacity to restore beiging in the LNR context (fig. S5, B to D). Collectively, these data support our conclusion that cold-induced CCL22 predominantly originates from M2 macrophages, and this induction is crucial for the M2-mediated beiging effects.

The SNS plays a critical role as an upstream regulator of cytokine secretion (*53*), and a very recent study demonstrates that the SNS can also regulate the secretion of IL-33 in inguinal LNs, thereby influencing beige adipocyte biogenesis (*33*). To examine the role of the SNS in LN-mediated CCL22 secretion, we used 6-hydroxydopamine (6-OHDA), a neurotoxic synthetic compound, to simultaneously block SNS function in the LNs and iWAT. We administered vehicle or 6-OHDA (10 mg/ml) directly into the iWAT and LNs of 10-week-old male C57BL/6 mice and subsequently exposed the mice to cold temperatures for 7 days before sorting M2 macrophages from iWAT or isolating and inducing M2 macrophages from bone marrow. We found that although 6-OHDA treatment inhibited iWAT beiging, as demonstrated by reduced UCP1 expression, it did not affect the release of CCL22 by M2 macrophages (fig. S6, A to C). The release of CCL22 in iWAT and bone marrow was also unaffected (fig. S6D). Together, these data support the notion that the release of CCL22 by M2 macrophages is largely not controlled by the SNS.

### Macrophage-derived CCL22 is obligatory for the cold-induced iWAT beiging

Since cold-induced expression of CCL22 in iWAT was abolished in the LNR group, we next examined if supplementing CCL22 protein could rescue the beiging defects of iWAT in the LNR group after cold exposure. First, we tested if a recombinant CCL22 (rCCL22) protein could promote beiging using our in vitro assay. SVF cells, which were derived from the 10-week-old Sham or LNR C57BL/6 mice, were pretreated with or without the rCCL22 protein (MDC, 10 ng/ml for 4 days) before differentiation into beige adipocytes. We found that replenishment with rCCL22 not only promoted sham-SVF beiging but also restored beige differentiation of the LNR SVF cells based on *Ucp1* mRNA expression and IF staining (Fig. 3, J and K). Consistent with these in vitro data, in vivo supplementing LNR mice with the rCCL22 protein (MDC, 20 μg/kg per day for 14 days) restored the cold-induced beiging in iWAT to a level comparable to that in sham mice, as determined by H&E, UCP1 IF staining, Western blotting, and qPCR of thermogenic genes (Fig. 3, L to N). Of note, supplementation with the rCCL22 protein in sham mice also promoted iWAT beiging in response to cold temperature compared to vehicle treatment (Fig. 3, L to N), which is consistent with our in vitro assay.

Next, we asked if CCL22-induced beige adipocyte formation was temperature-dependent. To test this, we repeated the above experiment at RT (fig. S7A), at which mice were only under mild to moderate cold stress. We found no substantial difference in body weight, blood glucose, core temperature, or food intake (fig. S7, B to E). Consistently, body fat mass, lean mass, and tissue weights had no changes compared to controls and rCCL22-treated groups (fig. S7, F to H). Even with increased levels of CCL22, there was no detectable change in UCP1 levels by IF staining and Western blotting, which was further confirmed by other thermogenic genes (fig. S7, I to L). In addition, rCCL22 did not promote beige adipocyte formation when treated with SVF cells at 37°C in vitro (fig. S7, M to O). These data suggest that without strong cold stimuli, CCL22 alone is not sufficient to promote iWAT beiging.

To probe whether CCL22 is a physiological regulator of iWAT beiging, we exposed 10-week-old C57BL/6 male mice to either 23° or 6°C for 14 days with bidaily subcutaneous injection of an anti-CCL22 neutralizing antibody to block the endogenous CCL22 (fig. S8A). We found that anti-CCL22 antibody injection had no effect on body weight, blood glucose, food intake, or lean mass but decreased core temperature and increased fat mass accompanied by a minor but not significant increase in the iWAT weight (fig. S8, B to H). As expected, upon cold exposure, thermogenic activity was induced in mice treated with the isotype control, as revealed by UCP1 IF staining and Western blotting together with qPCR of thermogenic genes (Fig. 3, O to R). However, these effects were diminished, with remaining beiging activity observed in mice treated with a CCL22-neutralizing antibody, as evidenced by the reduced induction of thermogenic genes and formation of beige adipocytes (Fig. 3, O to R). Our data support the notion that CCL22 signaling is essential for cold-induced iWAT beiging and thermogenesis.

### CCR4 is required for CCL22-promoted beige adipogenesis

**Fig. 4.**
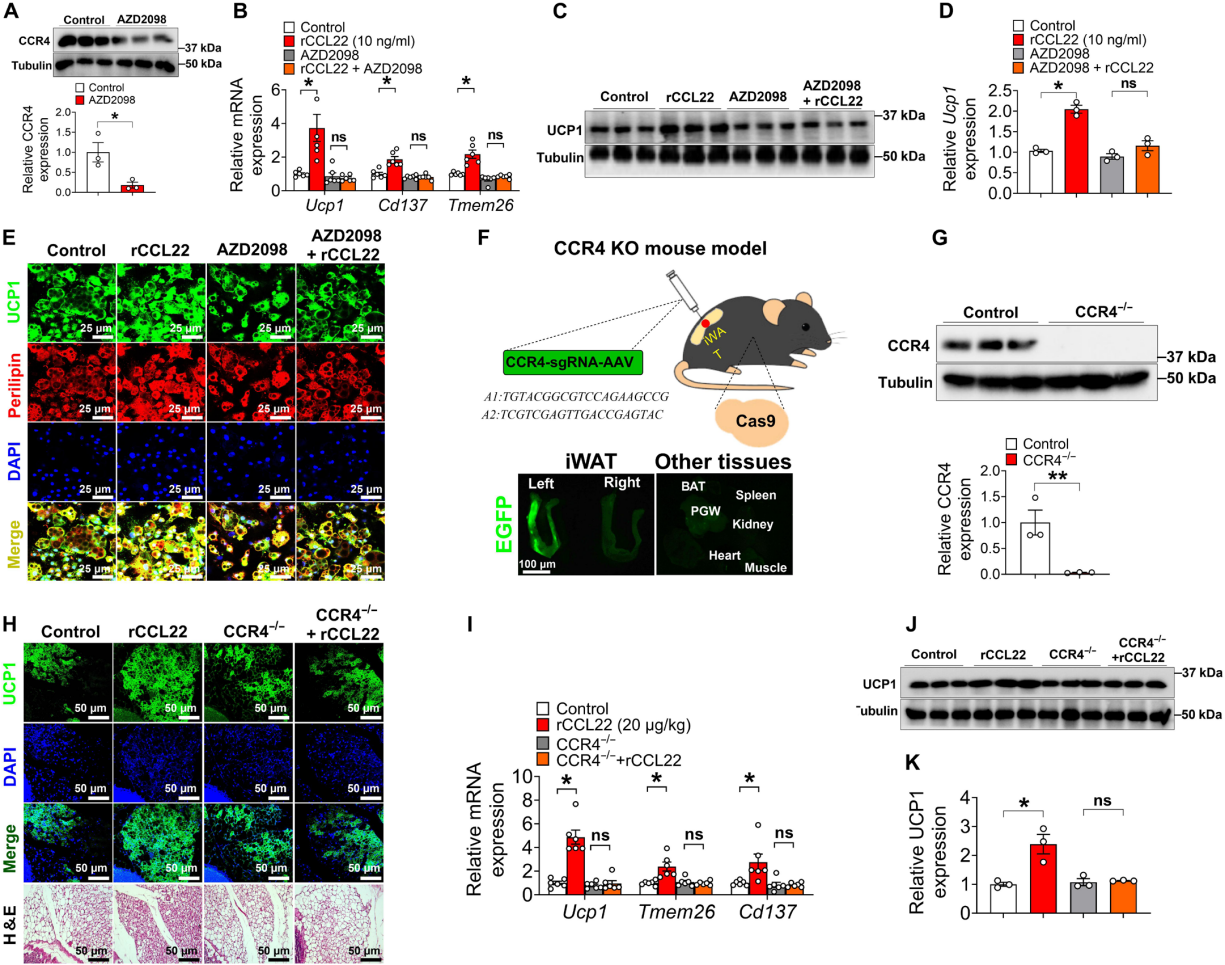
CCR4 is required for CCL22-induced iWAT beiging upon cold exposure. (**A**) Immunoblots and quantification of CCR4 in SVF cells. SVF cells from 10-week-old male C57BL/6 mice were treated with vehicle or AZD2098 (CCR4 antagonist, 100 nM) for 2 days (*n* = 3 per group). (**B**) mRNA expression of *Ucp1*, *Cd137*, and *Tmem26* in beige adipocytes. SVF cells from 10-week-old C57BL/6 male mice [divided into four groups: control, rCCL22 (10 ng/ml), CCR4 inhibitor (AZD2098), and AZD2098 + rCCL22] were first treated for 4 days and then induced to beige adipocytes for 5 days at 31°C. (**C** and **D**) Immunoblots (C) and quantification (D) of UCP1 (*n* = 3 per group). (**E**) Immunofluorescence of UCP1 in beige adipocytes. Scale bars, 25 μm. (**F**) Schematic representation of CCR4 KO mouse model generation by the CRISPR strategy. For 8-week-old mice, male Cas9 mice were iWAT-injected with AAV-sgRNAs-CCR4 [2 × 10^13^ genome copies/ml] to generate a CCR4 deletion mouse model (CCR4^−/−^). Enhanced green fluorescent protein (EGFP)–labeled AAV-sgRNAs-CCR4 virus was injected into the left iWAT of mice, and EGFP was not detected in the right iWAT and other tissues, verifying the successful injection of the virus. (**G**) Immunoblots and quantification of CCR4 in the iWAT SVF cells (*n* = 3 per group). Eight-week-old male Cas9 mice were injected with vehicle or AAV-sgRNA-CCR4 virus for 2 weeks. (**H** to **K**) Immunofluorescence of UCP1 and H&E staining in iWAT (H); mRNA expression of *Ucp1*, *Cd137*, and *Tmem26* in iWAT (I); and immunoblots and quantification of UCP1(J and K, respectively) (*n* = 3 to 6 per group). Eight-week-old male Cas9 mice were injected with vehicle or AAV-sgRNA-CCR4 virus for 2 weeks. Then, wild-type or CCR4 knockout mice were injected with vehicle or rCCL22 (20 μg/kg per day) for 2 weeks at 6°C. Scale bars, 50 μm. Data information: Results are presented as means ± SEM. [(A), (B), (D), (G), (I), and (K)] *P ≤ 0.05 and **P < 0.01 by nonpaired Student’s *t* test. ns, not significant.

Our data demonstrate that increasing CCL22 activity promotes iWAT beiging while inhibiting CCL22 diminishes iWAT beiging. It remained to be determined what downstream events mediated CCL22 in the beiging process. Since CCR4 is the sole receptor identified to date for CCL22 (*47*), we tested if CCR4 was involved in CCL22 mediated iWAT beiging. We pretreated SVF cells with vehicle [5% dimethyl sulfoxide (DMSO)] or AZD2098 (100 nM), a known CCR4 antagonist (*54*), for 2 days and then treated them with either vehicle or rCCL22 (MDC, 10 ng/ml) for 4 days. Cells were then induced to produce beige adipocytes for 5 days at 31°C. We found that AZD2098 treatment reduced CCR4 protein levels in SVF cells as previously reported (Fig. 4A). AZD2098 pretreatment completely abolished the effect of CCL22 on SVF beiging, as evidenced by mRNA levels, Western blotting, and UCP1 IF staining (Fig. 4, B to E). These data show that CCR4 antagonists can block CCL22-mediated effects on SVF beiging. AZD2098 alone did not impair SVF beiging ability (Fig. 4, B to E), implying the existence of CCL22/CCR4-independent pathways contributing to beiging.

To further investigate the role of CCR4 in CCL22-mediated beiging, we generated a CCR4 knockout (KO) mouse model using the CRISPR strategy. Specifically, 8-week-old male spCas9 mice, which have constitutive expression of CRISPR–associated protein 9 (Cas9) endonuclease directed by a CMV immediate enhancer/β-actin (CAG) promoter, were injected with an adeno-associated virus (AAV8) virus vector carrying paired single-guide RNAs (sgRNAs) targeting CCR4 gene (AAV8-sgRNAs-CCR4) into the iWAT to generate a CCR4 deletion mouse model (CCR4^−/−^) (Fig. 4F). Littermate spCas9 male mice receiving iWAT injections of AAV-vehicle virus served as the wild-type (WT) control group. AAV8 has been reported to effectively target iWAT (*55*, *56*). As expected, a high level of enhanced green fluorescent protein fluorescence was observed in the left injected side of iWAT, but not in the right non-injected side. No transgene expression was found in nearby or other organs, including muscle, heart, kidney, spleen, perigonadal white adipose tissue, and brown adipose tissue (Fig. 4F). As anticipated, we confirmed that CCR4 protein levels were entirely abolished in CCR4^−/−^ SVF cells (Fig. 4G). To test whether CCR4 mediates CCL22-induced beiging, after a 2-week AAV injection period, both WT and CCR4^−/−^ mice at postnatal day 70 (P70) were administered either vehicle or rCCL22 at a dosage of 20 μg/kg per day for 14 days 6°C (fig. S9A). In agreement with the anti-CCL22 data, rCCL22 treatment did not affect body weight, blood glucose levels, food intake, or lean mass but led to an increase in core temperature and a decrease in fat mass accompanied by a reduction in iWAT weight and enhanced energy expenditure in WT mice, but not in CCR4^−/−^ mice (fig. S9, B to L). Notably, CCR4 deletion largely diminished the effect of CCL22 on iWAT beiging, as evidenced by UCP1 IF staining, Western blotting, and qPCR analysis of thermogenic genes (Fig. 4, H to K). In line with this, CCR4 deletion impaired CCL22-mediated SVF beiging in vitro (fig. S9, M to O). Similar to the results observed with CCR4 inhibitor treatment, iWAT and SVF beiging capacity were not affected by CCR4^−/−^ (Fig. 4, H to K, and fig. S9, M to O). Collectively, these findings indicate that CCR4 is essential for mediating CCL22-induced beiging and energy metabolism in response to cold exposure.

### The CCL22/CCR4 axis promotes iWAT beiging by recruiting eosinophils into iWAT

**Fig. 5.**
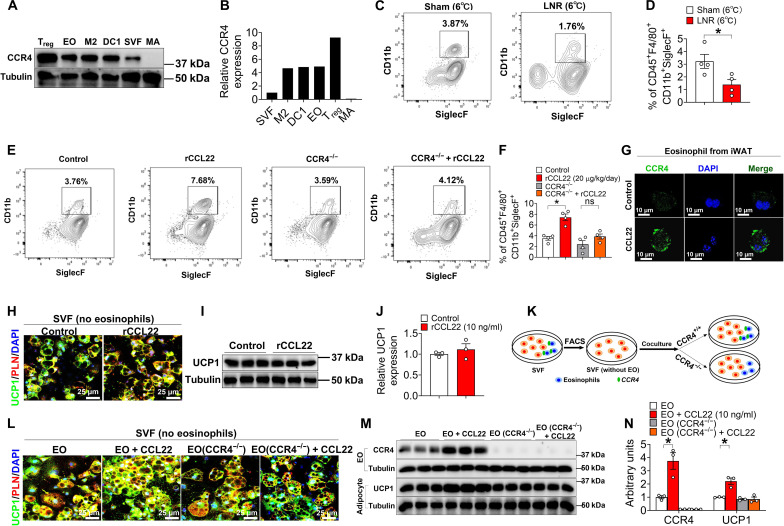
CCL22-promoted beiging is contingent upon the presence of CCR4 in eosinophils. (**A** and **B**) Immunoblots and quantification of CCR4 in T_reg_ cells (CD4^+^CD25^+^CD127^+^FoxP3^+^), eosinophils (EO; CD45^+^F4/80^+^CD11b^+^SiglecF^+^), M2 macrophages (M2; CD45^+^CD64^+^F4/80^+^CD206^+^), dendritic cells (DC; CD11c^+^I-A/I-E^+^CD103^+^), SVF cells, and MAs, isolated from five 10-week-old male C57BL/6 mice iWAT by flow cytometry sorting, respectively. (**C** and **D**) Flow cytometry analysis for eosinophils in iWAT from 10-week-old C57BL/6 male mice receiving sham or LNR for 7 days at 6°C (*n* = 4 per group). (**E** and **F**) Flow cytometry analysis of eosinophils in iWAT from 10-week-old male mice (*n* = 4 per group). Littermate control or CCR4 knockout mice were injected with vehicle or rCCL22 (20 μg/kg per day) into bilateral iWAT for 14 days at 6°C. (**G**) Immunofluorescence of CCR4 in eosinophils sorted from (E) and (F). Scale bars, 10 μm. (**H** to **J**) Immunofluorescence (H), immunoblots (I), and quantification (J) of UCP1 in beige adipocytes (*n* = 3 per group). Eosinophils from the iWAT SVF cells were sorted, treated with vehicle or rCCL22 (10 ng/ml) for 4 days at 31°C, and subsequently induced to differentiate into beige adipocytes for 5 days. Scale bar, 25 μm. (**K**) Schematic representation picture. Eosinophils (from BMDEs, transferred with vehicle or AAV-sgRNA-CCR4 virus for 3 days) and iWAT SVF cells (sorted out eosinophils) were cocultured for 2 days, treated with vehicle or rCCL22 for 4 days at 31°C and induced to form beige adipocytes for 5 days. SVF cells were obtained from 10-week-old Cas9 male iWAT depots. Eosinophils were obtained from the bone marrow of 10-week-old Cas9 male mice. (**L** to **N**) Immunofluorescence (L) of UCP1, immunoblots (M), and quantification (N) of CCR4 and UCP1 in eosinophils or beige adipocytes (*n* = 3 per group). Scale bars, 25 μm. Data information: Results are presented as means ± SEM. [(D), (F), and (N)] *P ≤ 0.05 by nonpaired Student’s *t* test.

Next, we wanted to further delineate the cellular targets of CCR4 in iWAT. We found that CCR4 was highly enriched in adipose regulatory T cells (T_regs_), eosinophils, M2 macrophages, DCs, and SVF cells, but not in MAs (Fig. 5, A and B). Among these cell types, we observed the decrease of eosinophils (CD45^+^CD11b^+^F4/80^+^Siglec-F^+^) by cold stimuli in the LNR group (Fig. 5, C and D). In addition, the number of eosinophils was proportionally increased by cold stimuli, and this accumulation was diminished by anti-CCL22 antibody treatment (fig. S10, A to C). Consistent with this, cold-stimulated eosinophil markers, including SiglecF and interleukin-4 (IL-4), were abolished by anti-CCL22 treatment (fig. S10, D and E). Furthermore, rCCL22 treatment (iWAT injection of 10-week-old C57BL/6 mice housed at 6°C, 20 μg/kg per day) recruited more eosinophils in iWAT (Fig. 5, E and F), accompanied by the increased CCR4 expression in eosinophils (Fig. 5G). Moreover, this CCL22-induced recruitment could be reversed in CCR4 KO mice (Fig. 5, E and F). These results suggest that CCL22 recruits eosinophils through its receptor CCR4 in iWAT in response to cold stimuli.

Using SVF cells without eosinophils, we next investigated the role of eosinophils in CCL22-mediated beiging. We found that the CCL22-enhanced beiging effect was no longer present in SVF cells without eosinophils (Fig. 5, H to J). In addition, replenishment of cultured bone marrow–derived eosinophils (BMDEs) restored CCL22-mediated beiging, but this restoration was impaired when eosinophils were depleted of CCR4 (Fig. 5, K to N). Together, these data support a model in which CCR4 in eosinophils is required for CCL22 mediated beiging in iWAT.

T_H_2 cells, which also express CCR4, have been implicated in the thermogenic program in iWAT (*49*). To clarify whether T_H_2 cells play a role in the observed effects mediated by CCR4, we set up cocultures of SVF cells (which lack T_H_2 cells) with CCR4-deficient T_H_2 cells. We sourced the T_H_2 cells (CD45^+^ CD4^+^ FOXP3^−^ GATA3^+^ T1/ST2^+^) from the bone marrow of 10-week-old Cas9 male mice and then treated these cocultures with CCL22 (fig. S11A). Our findings indicate that in the absence of T_H_2 cells, CCL22-induced UCP1 and CCR4 levels remain unaffected (fig. S11, B and C). Moreover, CCL22 still promotes beiging, as evidenced by UCP1 expression, even in CCR4-deficient T_H_2 cells (fig. S11, D and E).

In addition to T_H_2 cells, T_reg_ cells have previously been identified as modulators of iWAT beiging (*57*). We found the number of T_reg_ cells (CD4^+^CD25^+^CD127^+^FoxoP3^+^) was reduced in the LNR group (fig. S12, A and B). Since CCR4 is most highly expressed in T_reg_ cells (Fig. 5, A and B), we asked whether T_regs_ expressing CCR4 mediated CCL22-induced beiging phenotypes. To test this, we removed the T_reg_ portion from the SVF cells by sorting and treating them with rCCL22. We observed similar CCL22-mediated promoting effects on beiging, indicating that T_reg_ cells are not required for CCL22-mediated beiging (fig. S12, C to E). Of note, SVF cells without T_reg_ cells had slightly reduced beiging potential in vitro (fig. S12, C to E). These results indicate that T_reg_ cells are not required for CCL22-induced beige adipocyte formation. These results collectively highlight that CCL22-induced beige adipocyte formation is predominantly independent of both T_H_2 and T_reg_ cells and CCR4 expression within them.

### The FAK/p65 pathway in eosinophils is required for CCL22-mediated iWAT beiging

**Fig. 6.**
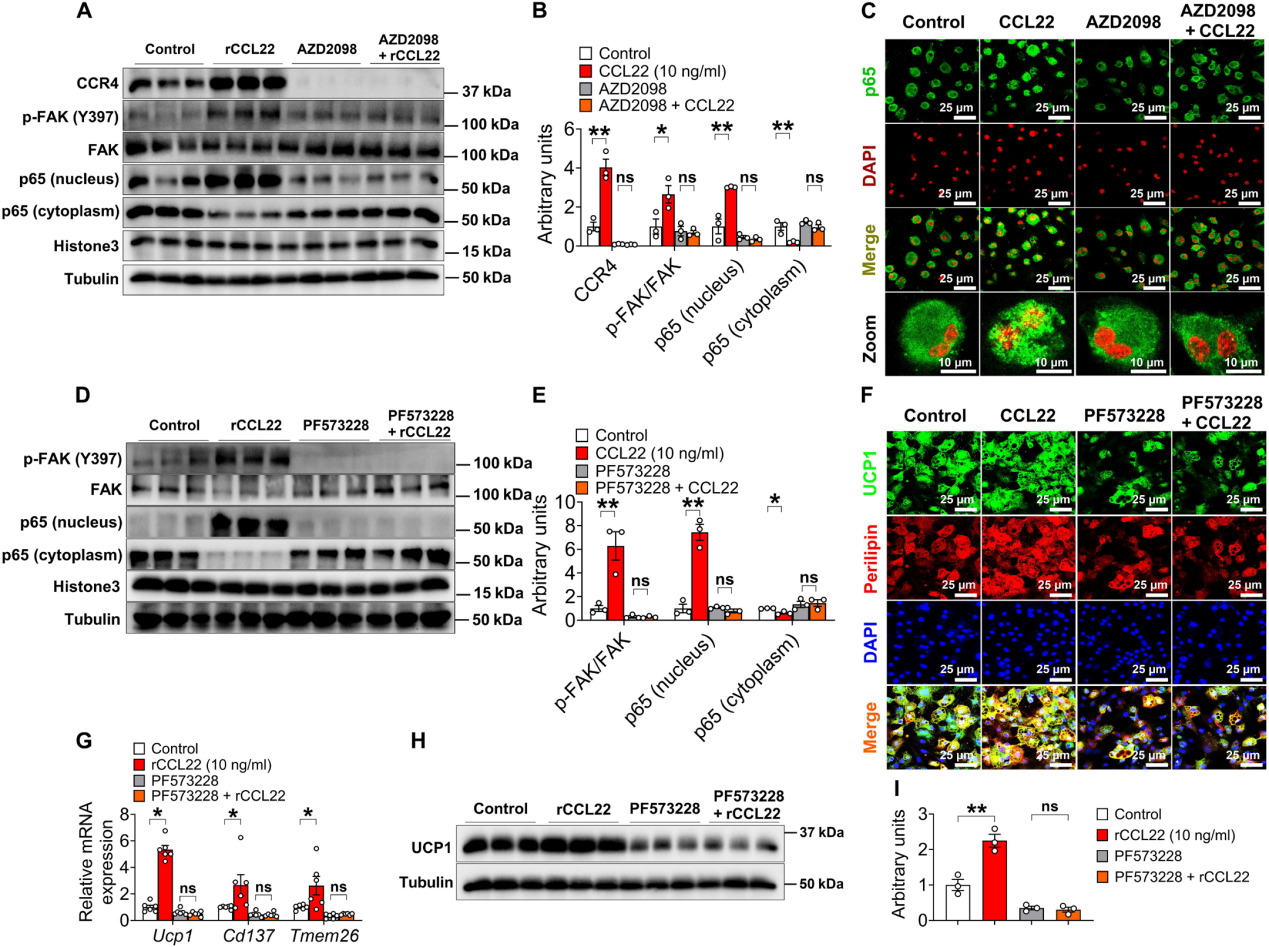
The FAK/p65 pathway in eosinophils is required for CCL22-promoted beiging. (**A** and **B**) Immunoblots (A) and quantification (B) of CCR4, p-FAK, and p65 protein in eosinophils cultured with a vehicle, AZD2098 (CCR4 inhibitor, 100 nM), rCCL22 (10 ng/ml), or AZD2098 + rCCL22 (10 ng/ml) for 48 hours (*n* = 3 per group). Eosinophils were isolated and induced from BMDEs of 10-week-old male C57BL/6 mice receiving 6°C stimulation for 14 days. (**C**) p65 translocation in eosinophil cells cultured with vehicle, AZD2098 (100 nM), rCCL22 (10 ng/ml), or AZD2098 + rCCL22 (10 ng/ml) for 48 hours (*n* = 3 per group). Scale bars, 10 μm. (**D** and **E**) Immunoblots (D) and quantification (E) of p-FAK and p65 protein in eosinophils cultured with vehicle, PF5733228 (FAK inhibitor, 5 μM), rCCL22 (10 ng/ml), or PF5733228 + rCCL22 (10 ng/ml) for 48 hours. (**F** to **I**) Immunofluorescence (F) of UCP1, mRNA expression of *Ucp1* (G), immunoblots (H), and quantification of UCP1 (I) in beige adipocytes (*n* = 3 per group). Eosinophils (treated with vehicle or PF573228 for 2 days) and iWAT SVF cells (without eosinophils) were cocultured for 4 days, and beige adipocytes were then induced for 5 days at 31°C. SVF cells were obtained from 10-week-old C57BL/6 male mouse iWAT. Eosinophils were obtained from the BMDMs of 10-week-old C57BL/6 male mice. Scale bar, 25 μm. Data information: Results are presented as means ± SEM. [(B), (E), (G), and (I)] *P ≤ 0.05 and **P < 0.01 by nonpaired Student’s *t* test.

To investigate how CCL22/CCR4 induced the intracellular signaling pathway(s) of eosinophils, we tested the phosphorylation of the focal adhesion kinase (FAK) pathway, which has been reported to be involved in eosinophil migration and adipose beiging (*13*, *58*). We found that CCL22 elevated the levels of CCR4 and the phosphorylation of FAK within BMDEs, and both were abolished by CCR4 antagonist AZD2098 treatment (Fig. 6, A and B). We then tested a key downstream event induced by phosphorylation of FAK: nuclear factor κB (NF-κB) p65 translocation. Consistent with the increased phosphorylation of FAK, we found that CCL22 promoted the nuclear localization of p65 within eosinophils, and this phenotype was also abolished by AZD2098 treatment (Fig. 6, A to C). These data suggest that CCL22 promotes phosphorylation of FAK and NF-κB p65 translocation via CCR4 within eosinophils.

To test the involvement of the intracellular signaling molecules FAK in CCL22-mediated beiging, a selective FAK inhibitor PF573228 was used. As expected, preincubation of eosinophils with PF573228 for 2 days before coculture with SVF cells abolished the phosphorylation of FAK (Fig. 6, D and E). We then cocultured eosinophils with SVF with or without CCL22. PF573228 completely abolished NF-κB p65 nuclear translocation (Fig. 6, D and E) and the CCL22-mediated up-regulation of thermogenic gene mRNA expression and *Ucp1* protein expression without noticeable cytotoxicity (Fig. 6, F to I). Of note, PF573228 alone slightly but not significantly reduced the beiging phenotype (Fig. 6, F to I). Together, these data suggest that the FAK/p65 pathway in eosinophils is required for CCL22/CCR4-mediated beiging.

### SMA^+^ APC cells contribute to CCL22-induced beige adipogenesis

Beige adipocytes can be generated through de novo recruitment from APCs or through trans-differentiation from white adipocytes (*5*–*14*, *17*). Moreover, we have recently reported that UCP1^+^ beige adipocytes can also act as a cellular source for iWAT beiging around the LNs (*28*). Our data thus far suggest that SVF APCs are the primary cellular targets of CCL22-induced beige adipocytes. To test whether beige adipocytes contributed to CCL22-mediated beiging, we first induced SVF cells into beige adipocytes and then incubated them with rCCL22 (10 ng/ml) for 5 days at 31°C (fig. S13A). Cells were subsequently harvested to assess beige adipocyte quantity and cellular respiration activity. Our data demonstrated that, unlike SVF cells, CCL22 treatment in beige adipocytes did not further enhance beige adipocyte formation (fig. S13, B and C). We then investigated whether mature white adipocytes contribute to CCL22-induced beige adipocyte formation. We differentiated SVF cells into white adipocytes and treated them with rCCL22 for 5 days at 31°C, similar to the treatment of beige adipocytes (fig. S13D). However, no differences in beiging capability were observed between the control group and the rCCL22-treated group (fig. S13, E and F). Consistent with the beiging levels, the OCR of beige adipocytes was higher than that of control cells when SVF cells, but not MAs, were treated with rCCL22 (fig. S14, A to H). Notably, the effect of CCL22 on OCR was only significant when the SVF cells were challenged with cold temperature (31°C) but not warm temperature (37°C) (fig. S14, A to H). Overall, these findings indicate that CCL22 primarily exerts its beiging effects on SVF cells, rather than on white or beige adipocytes.

**Fig. 7.**
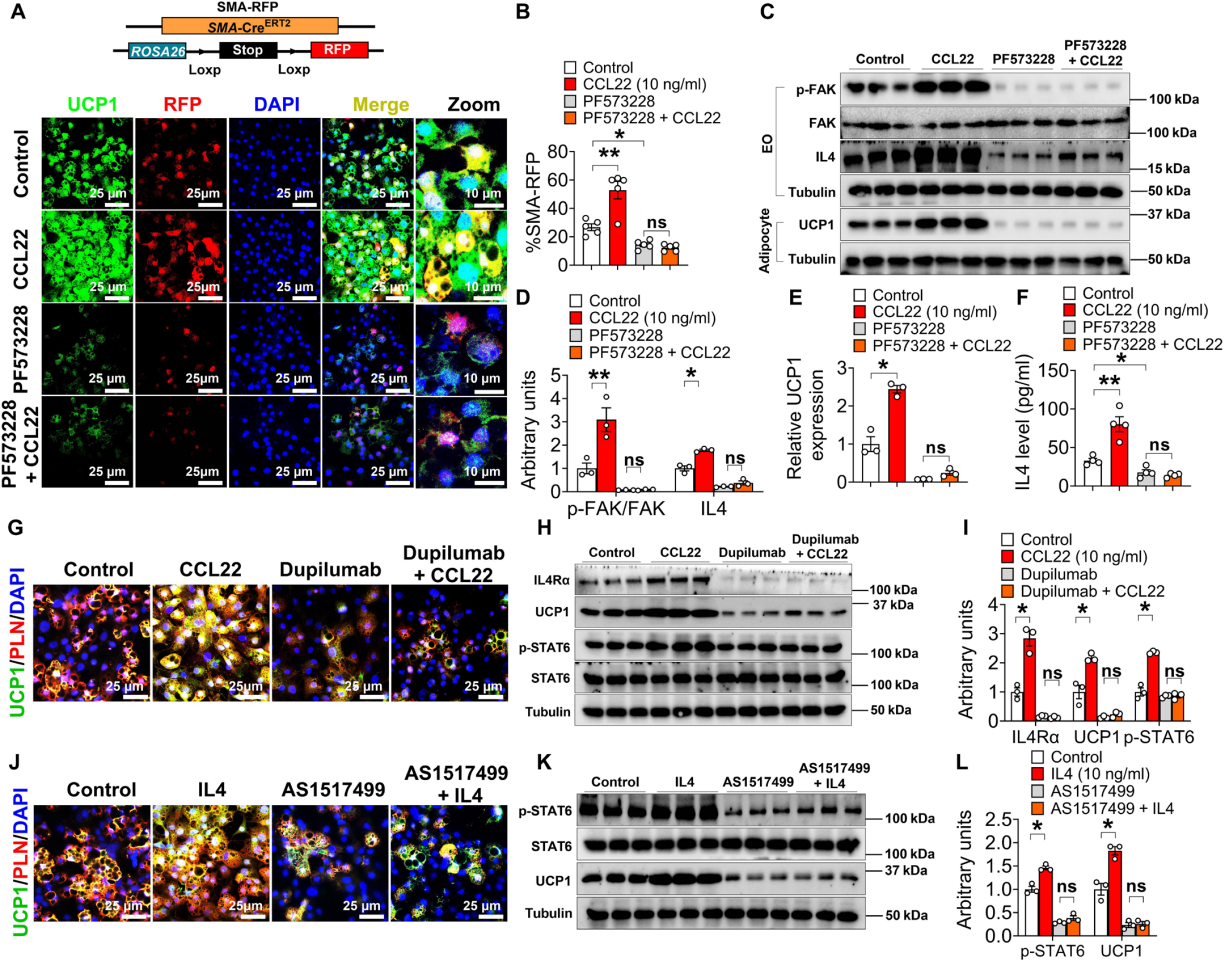
IL-4 signaling mediates the FAK/p65 signaling in eosinophils to promote SMA^+^ SVF beiging. (**A**) Immunofluorescence of UCP1 and RFP in SMA-RFP^+^–derived beige adipocytes. Eosinophils were cocultured with iWAT SVF cells (excluding eosinophils) for 4 days, after which the SVF cells were induced to differentiate into beige adipocytes for 5 days at 31°C. SVF cells and eosinophils were obtained from the iWAT and induced from the bone marrow, respectively, of 10-week-old SMA-RFP^+^ male mice. During the coculture period, eosinophils were treated with vehicle, PF5733228 (FAK inhibitor, 5 μM), rCCL22 (10 ng/ml), or a combination of PF5733228 and rCCL22. Scale bars, 25 μm. (**B**) Quantification of the percentage of RFP^+^ cells that express UCP1 (*n* = 5 per group). (**C** to **E**) Immunoblots (C) and quantification [(D) and (E)] of p-FAK and IL-4 protein in eosinophils and UCP1 in beige adipocytes from (A). (**F**) IL-4 levels in the medium of eosinophils (*n* = 3 per group). Eosinophils were treated with vehicle, PF5733228 (5 μM), rCCL22 (10 ng/ml), or PF5733228 + rCCL22 for 4 days. (**G** to **I**) Immunofluorescence (G) of UCP1, immunoblots (H), and quantification (**I**) of UCP1 and IL-4Rα. iWAT RFP^+^ SVF cells were first sorted and then cultured with vehicle, dupilumab (IL-4Rα inhibitor, 50 μM), rCCL22 (10 ng/ml), or dupilumab + rCCL22 for 4 days, and then induced to beige adipocytes for 5 days at 31°C. (*n* = 3 per group). (**J** to **L**) Immunofluorescence (J) of UCP1, immunoblots (K), and quantification (L) of UCP1 and p-STAT6. iWAT RFP^+^ SVF cells were first sorted and then cultured with vehicle, AS1517499 (p-STAT6 inhibitor, 50 μM), rIL-4 (10 ng/ml), or AS1517499 + rIL-4 for 4 days, and then induced to beige adipocytes for 5 days at 31°C (*n* = 3 per group). Data information: Results are presented as means ± SEM. [(B), (D) to (F), (I), and (L)] *P ≤ 0.05 and **P < 0.01 by nonpaired Student’s *t* test.

Using selective genetic lineage tracing tools, iWAT Pdgfrα^+^ adventitia fibroblasts, Pdgfrβ^+^ pericytes, and Sma^+^ perivascular cells can emerge into beige adipocytes upon cold stimuli (*6*, *10*–*12*). To further investigate the cellular sources within SVF cells that can contribute to CCL22-induced beige adipocyte formation, we combined three different inducible Cre lines (Sma-Cre^ER^, Pdgfrα-Cre^ER^, and Pdgfrβ-Cre^ER^) with the indelible Rosa26-LSL-RFP (*R26^RFP^*) reporter mice to create Sma-RFP, PRα-RFP, and PRβ-RFP, respectively. Tamoxifen (TAM) was administered to these mice for two consecutive days, and SVF cells were isolated from iWAT for in vitro beige adipogenesis. As expected, CCL22 treatment promoted beige adipogenesis in all three settings, based on UCP1 IF staining and Western blotting (Fig. 7, A, C, and E; and fig. S15, A to H). However, we only observed a notable increase in RFP-labeled UCP1^+^ beige adipocytes from the Sma^+^ source, but not from Pdgfrα^+^ or Pdgfrβ^+^, as revealed by the percentage of RFP^+^ UCP1^+^ among all UCP1^+^ beige adipocytes (Fig. 7B, and fig. S15, A to H). Together, these data suggest that CCL22 promotes beige adipogenic potential primarily originating from Sma^+^ SVF cells.

### IL-4 signaling mediates the FAK/p65 signaling in eosinophils to promote SVF beiging

Next, to demonstrate the crucial role of FAK/p65 pathway activation in SMA-derived beige adipocyte activation, we added PF573228, a FAK inhibitor, to the sorted SMA-RFP^+^ SVF cells and performed beige adipogenesis. We found that PF573228 alone was able to block FAK activity, impair overall and SMA^+^-derived beige adipogenesis, as well as CCL22-promoted beige adipogenesis, as demonstrated by RFP and UCP1 IF staining and Western blotting (Fig. 7, A to E). These results provide further evidence that the FAK/p65 pathway is a critical mediator of beige adipocyte activation, particularly in the SMA^+^ subpopulation.

To understand how the activation of the FAK signaling pathway in eosinophils promotes beige adipogenesis, we examined changes in all cytokines and chemokines secreted by eosinophils. Among them, we found that interleukin-4 (*Il4*) and *Il13* mRNA in eosinophils were suppressed, but *Il13* protein expression showed no difference (fig. S15, I to K), indicating that FAK may regulate beige adipocyte formation through *Il4* expression. Moreover, CCL22 promoted *Il4* expression and secretion in eosinophils, but when FAK was inhibited in the presence of PF573228, CCL22 cannot promote *Il4* expression and secretion (Fig. 7, C to F), suggesting that CCL22 primarily regulates *Il4* through the FAK signaling pathway in eosinophils.

IL-4 is an important cytokine involved in the immune system, and it has been found to play an important role in promoting the formation of beige adipocytes, mainly through its receptor, IL-4Rα (IL-4 receptor alpha) (*35*, *59*, *60*). To investigate whether CCL22 regulates beige adipocyte formation through the IL-4 signaling pathway in eosinophils, we used dupilumab, an IL-4Ra inhibitor, to block the IL-4 receptor in sorted SMA^+^ SVF cells. We found that in the presence of dupilumab, CCL22 was no longer able to promote SMA^+^ beige adipogenesis (Fig. 7, G to I). These findings further underscore the critical role of IL-4 and its receptor, IL-4Rα, in CCL22-mediated beige adipocyte formation. How does IL-4 secreted by eosinophils regulate beige adipogenesis? IL-4 has been known to exert its biological effects via activation of the transcription factor signal transducer and activator of transcription 6 (STAT6) (*61*). In agreement, we found that IL-4 promoted p-STAT6 expression, and when p-STAT6 expression was inhibited (with AS1517499, a p-STAT6 inhibitor), IL-4 no longer promoted beige adipogenesis (Fig. 7, J to L), indicating that IL-4 secreted by eosinophils mainly regulates beige adipogenesis through the p-STAT6 pathway. Together, CCL22 primarily acts on the FAK/p65 signaling pathway in eosinophils to promote IL-4 release, and IL-4 regulates the p-STAT6 pathway through the IL-4Rα in Sma^+^ APCs, ultimately promoting beige adipocyte formation (fig. S16).

rCCL22 can promote the SVF beiging in vitro, indicating the presence of eosinophils in culture. When we removed eosinophils or ablated CCR4 in eosinophils before coculturing with SVF cells, rCCL22 lost its ability to promote SVF beiging. To our surprise, our flow data suggested the presence of eosinophils even in the Sma^+^ SVF cells, and this number was notably higher when rCCL22 was present (fig. S17A). The presence of eosinophils in culture was further supported by the measurement of IL-4 levels in the medium 2 and 4 days after culturing (fig. S17B). In line with the observation that rCCL22 enhances the count of eosinophils, IL-4 levels were increased by rCCL22 treatment in our culture system (fig. S17B).

### CCL22 prevents HFD-induced obesity and promotes beiging in both mice and humans

**Fig. 8.**
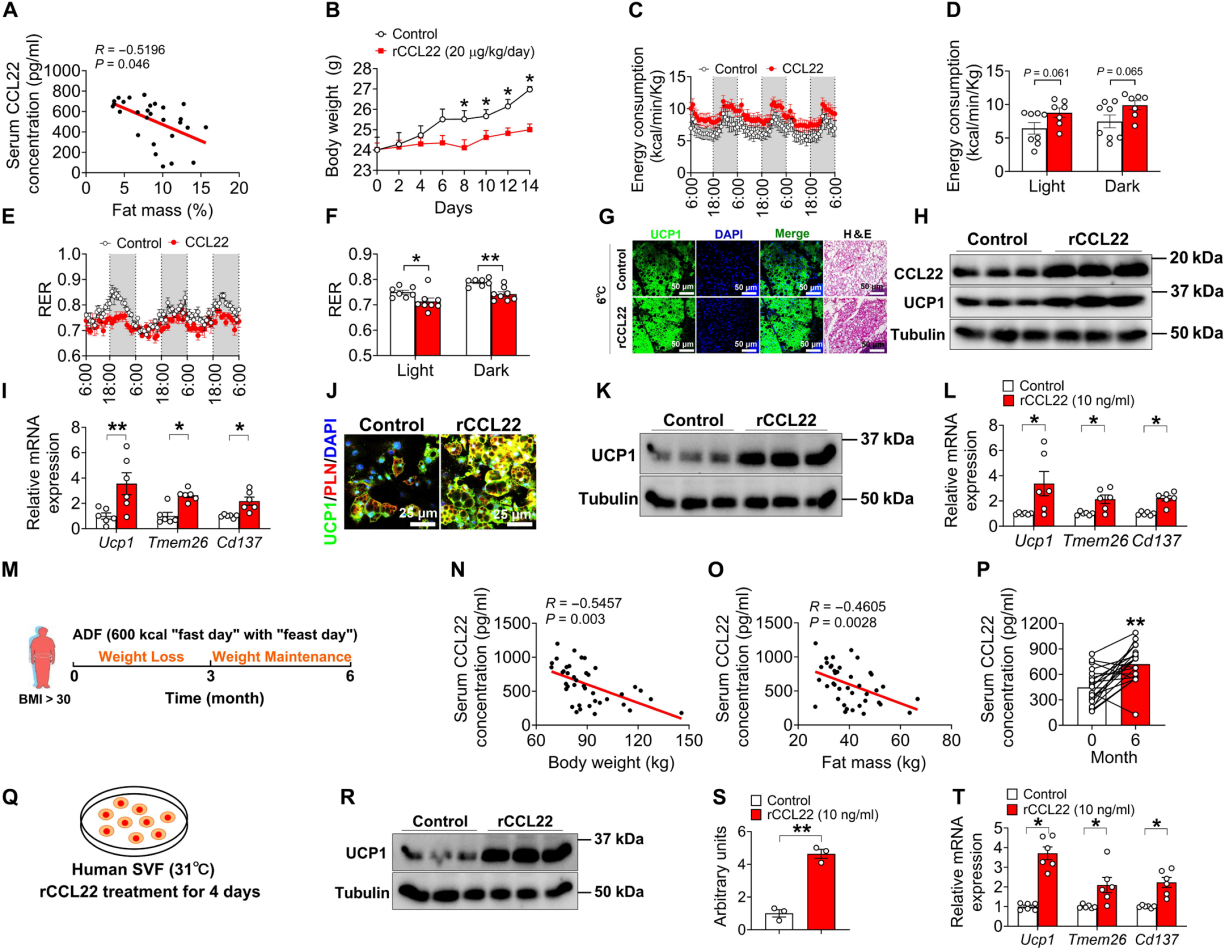
CCL22 prevents HFD-induced obesity and promotes beiging both in mice and humans. (**A**) Pearson’s correlation coefficient analysis of serum CCL22 concentration and fat mass (*n* = 28 per group). (**B** to **F**) Body weight (B), energy consumption rate [(C) and (D)], and RER [(E) and (F)] (*n* = 8 per group). Ten-week-old C57BL/6 male mice received saline or rCCL22 (20 μg/kg per day) at 6°C for 14 days with HFD. (**G** to **I**) Immunofluorescence (G), immunoblots (H) of UCP1, and mRNA expression (I) of thermogenic genes in iWAT (*n* = 3 to 6 per group). Scale bar, 50 μm. (**J** to **L**) Immunofluorescence (J), immunoblots, and quantification [(K) and (L)] of UCP1 in iWAT (*n* = 3 per group). SVF cells were extracted from HFD-fed iWAT, cultured with vehicle or rCCL22 (10 ng/ml) for 4 days, and then induced to beige adipocytes for 5 days. Scale bars, 25 μm. (**M**) Schematic diagram: The 6-month trial of obese adults participated in ADF: a 3-month weight loss period followed by a 3-month weight maintenance period. (**N** and **O**) Pearson’s correlation coefficient analysis of human serum CCL22 levels, body weight (N), and fat mass (O) (*n* = 40). (**P**) Human serum CCL22 levels (*n* = 20). (**Q**) Schematic diagram: Human SVFs from iWAT were cultured at 31°C for 5 days, treated with vehicle or CCL22 (10 ng/ml) for 4 days, and then induced to beige adipocytes. (**R** to **T**) Immunoblots and quantification [(R) and (S)] of UCP1 and mRNA expression (T) of thermogenic genes in human beige adipocytes (*n* = 3 to 6 per group). Data information: Results are presented as means ± SEM. [(A), (N), and (O)] Two-tailed Pearson’s correlation coefficient analysis. [(F), (I), (L), (S), and (T)] *P ≤ 0.05 and **P < 0.01 by nonpaired Student’s *t* test. (P) **P < 0.01 by nonpaired Student’s *t* test compared with month 0 body weight. (B) *P ≤ 0.05 by two-way analysis of variance (ANOVA) followed by post hoc Bonferroni tests.

Since our data have shown that CCL22 can act as a beiging agent, we reasoned that its expression might be reduced in obese adipose tissues. We observed that serum CCL22 levels were negatively associated with body fat in mice (Fig. 8A). To explore the potential impact of CCL22 in adiposity and energy metabolism, 10-week-old C57BL/6 mice were iWAT-injected with vehicle or rCCL22 (20 μg/kg per day) for 14 days while also undergoing cold exposure (6°C) and HFD treatment (fig. S18A). The results showed that rCCL22 supplementation had several beneficial effects on the treated mice. rCCL22 supplementation increased the core temperature of the mice, reduced their blood glucose levels, and prevented body weight and body fat gain upon HFD treatment without any differences in food intake (Fig. 8B and fig. S18, B to G). These findings suggest that CCL22 may play a role in regulating energy expenditure, contributing to the reduction in body weight and body fat gain.

In agreement with reduced body weight and body fat gain, CCL22-treated mice on an HFD trended toward slightly higher whole-body energy expenditure compared with controls upon cold exposure, with no difference observed in locomotor activity (Fig. 8, C and D, and fig. S18, D, H, and I). CCL22 treatment reduced the RER (Fig. 8, E and F), indicating a relative increase in lipid oxidation for the breakdown of fats to generate energy. Consistent with the observed increase in energy expenditure, we found that CCL22 enhanced cold-induced UCP1 expression in iWAT from HFD-fed mice (Fig. 8, G to I). In addition, supplementation of the rCCL22 protein in vitro with SVF cells isolated from HFD-fed iWAT also promoted beige adipogenesis based on UCP1 IF staining and Western blotting (Fig. 8, J to L). Next, we assessed the impact of enhanced thermogenic adipocytes on systemic glucose metabolism by measuring glucose tolerance and insulin sensitivity. HFD-fed mice supplemented with rCCL22 supplementation showed marked improvement in their glucose tolerance, as measured by the glucose tolerance test (GTT) (fig. S18, J and K). In contrast, insulin tolerance was slightly improved, but not significantly different, in rCCL22 mice compared to control mice after 2 weeks of HFD feeding (fig. S18, L and M). Thus, our mouse data suggest that rCCL22 supplementation combined with cold exposure protects mice from DIO and its associated metabolic impairments.

We then asked if there was a translational perspective in humans based on our mouse studies. Taking advantage of a previously established obesity and weight loss study (*62*–*64*), which was alternate day fasting (ADF) combined with a low carbohydrate diet (30% carbohydrates, 35% protein, and 35% fat), we asked if CCL22 played a role in obesity and ADF-mediated weight loss (Fig. 8M). We first assessed CCL22 protein levels in the blood of a cohort of obese adults [*n* = 40, body mass index (BMI) > 30 kg/m^2^] using a CCL22/MDC ELISA. Our findings revealed a negative correlation between body weight, fat mass percentage, and CCL22 levels in the blood (Fig. 8, N and O). In addition, we noticed that among those who lost weight during a 6-month ADF period, CCL22 levels were significantly increased (*n* = 20; Fig. 8P). Last, we explored the role of CCL22 in promoting SVF beiging from human adipose tissue. We isolated SVF cells from human subcutaneous adipose tissues (SATs) and cultured them with or without rCCL22 for 4 days. This was followed by exposure to cold (31°C) for 20 days to induce beiging (Fig. 8Q). We observed increases in UCP1 level and the expression of other thermogenic genes in CCL22-treated obese human SVF cells (Fig. 8, R to T). These data suggest that the implementation of CCL22 promotes the formation of beige adipocytes and supports its positive impact on energy metabolism. Our data collectively show that decreased CCL22 signaling in humans may contribute to the impairment of WAT beiging and the development of obesity.

## DISCUSSION

Despite extensive research on iWAT beiging in recent years, the mechanisms underlying the anatomic location of this process remain poorly understood. In the present study, we reveal that MDC CCL22, a protein whose expression increases in response to cold and decreases in obesity, acts as a positive regulator of local LN-mediated beiging of iWAT and thermogenesis. First, reducing CCL22 signaling impairs cold-induced iWAT beiging and thermogenesis, and activating CCL22 signaling can promote beiging and thermogenesis. In addition, we show that CCL22 exerts its effects by binding to its receptor CCR4, which attracts eosinophils into iWAT, resulting in the stimulation of iWAT beiging in response to cold exposure. Last, our clinical studies reveal that obese individuals have lower serum levels of CCL22 compared to non-obese controls, and weight loss interventions lead to an increase in serum CCL22 levels. Consistent with its anti-obesogenic role, activating CCL22 mitigates the development of DIO. Together, our results from human and mouse studies demonstrate that CCL22 plays a role in promoting iWAT beiging, maintaining glucose homeostasis, and boosting total energy expenditure. As a result, the CCL22/CCR4 axis represents a promising therapeutic target for preventing the development of obesity and other metabolic disorders, such as insulin resistance.

Thermogenesis is crucial in energy homeostasis, and recent studies have suggested that thermogenesis is regulated via a complex interplay between APCs and immune cells, notably macrophages (*65*, *66*). For example, earlier studies proposed that iWAT macrophages could promote iWAT beiging by producing catecholamines (*67*). However, a recent multi-laboratory report challenges this notion by showing that macrophages do not express tyrosine hydroxylase and, as a result, cannot directly produce catecholamines (*68*). In addition, a subset of macrophages associated with sympathetic neurons has been found to metabolize norepinephrine, which could potentially decrease sympathetic stimulation of adipose tissue and thereby hinder thermogenesis (*69*). Despite these findings, the important role of M2 macrophages in iWAT beiging has been reinforced by multiple studies. For instance, studies have shown that the secretion of slit guidance ligand 3 from M2 macrophages can stimulate sympathetic innervation, leading to iWAT beiging (*41*). Consistent with this notion, another study found that IL-25–induced shifts in macrophage polarization can promote APC proliferation and beige adipocyte development (*42*). In addition to M2 macrophages, eosinophils are also thought to be necessary for cold-induced beiging of iWAT by promoting the polarization of M2-like macrophages (*34*, *59*). Both WAT eosinophils and M2-like macrophages decline with weight gain associated with metabolic impairments (*70*). Thus, these findings suggest an immune-APC circuit to ensure the appropriate WAT remodeling to meet different metabolic demands. Our findings have substantiated the important role of M2 macrophages and eosinophils in cold-induced iWAT beiging and identified an important player, CCL22, and its receptor CCR4, linking the two cell types. Notably, our findings differ from previous studies in that we show that M2 macrophages play a key role in the upstream regulation of eosinophils: M2 macrophages secrete CCL22, which attracts eosinophil accumulation into iWAT by increased CCR4 levels in response to cold exposure. Together, these findings support the notion that there is a previously unidentified positive feedforward loop between M2 macrophages and eosinophils, collectively creating an immune microenvironment favorable for iWAT beiging.

From a mechanistic perspective, we have revealed the eosinophil CCR4 pathway as a downstream event that cooperates with M2 macrophage–derived CCL22 to govern the activity of APCs for beiging. Upon activation of CCL22, CCR4 expression in eosinophils is increased, which leads to more eosinophil recruitment into iWAT, accompanied by activation of FAK/p65 activation and iWAT beiging. In addition to eosinophils, CCR4 is expressed in various T cell subsets such as T_reg_ cells, which have previously been identified as modulators for iWAT beiging (*35*, *60*). Thus, CCR4 expressing T_reg_ cells may mediate CCL22-induced beiging. Toward this end, we analyzed SVF cells without T_reg_ cells. CCL22 can still promote SVF beiging in the absence of T_reg_ cells. Thus, our data suggest that the contribution of CCR4 is primarily mediated in eosinophils but not T_reg_ cells in mediating CCL22-induced beiging.

Previous studies have shown that beige adipocytes can arise through de novo differentiation from resident APCs or conversion of mature white adipocytes (*5*–*14*, *17*). Our findings suggest that the CCL22-CCR4 axis promotes beige adipogenesis through APCs, rather than mature white adipocytes. Among several cellular sources of beige APCs, including Sma^+^, Pdgfrα^+^, and Pdgfrβ^+^ cells, we determined that CCL22-induced beige adipocytes are predominantly derived from Sma^+^ SVF cells, whereas Pdgfrα^+^ and Pdgfrβ^+^ SVF cells do not contribute to this process. The exact signaling pathway within Sma^+^ APCs that facilitates the link between CCR4-activated FAK/p65 signaling in eosinophils and Sma^+^ APCs, a key process for the beiging of iWAT triggered by cold exposure, is yet to be determined. Nevertheless, our extensive in vivo and in vitro data strongly indicate that the recruitment of eosinophils, orchestrated by CCL22, along with the ensuing CCR4 activity, plays pivotal roles in the process of CCL22-induced beiging in iWAT. However, whether CCL22 directly affects eosinophils through CCR4 binding remains uncertain. It has been suggested before that exogenous CCL22 can prompt eosinophil migration, yet endogenous CCL22 does not seem to influence eosinophil movement during allergic inflammation (*71*). However, other research shows the ability of CCL22 to recruit eosinophils (*72*).

Compared to the origins of APCs, the origins and mechanisms of macrophages that emerge during cold exposure remain less clear. Since other subsets of macrophages in adipose tissue can impede adaptive thermogenesis and elicit the pathogenesis of obesity, a better understanding of the origins of macrophages during cold exposure is an important area of research in the field (*37*, *38*). To address these questions, available macrophage lineage tracing mouse models, such as Csf1R-Cre^ER^; R26^RFP^ and CX3CR1-Cre^ER^; R26^RFP^, can be used. Csf1R-RFP mice can track all adipose macrophages, including M1- and M2-like macrophages, while CX3CR1-RFP mice can trace the monocyte-derived macrophages through TAM treatment in the adult stage. Mechanistically, several prior studies have demonstrated that cold-induced M2 macrophage accumulation within iWAT arises from enhanced proliferation of M2 cells rather than a transition from M1 to M2 polarization (*44*). Consistent with this, our flow cytometry results and the absence of changes in M1 marker expression upon cold exposure suggest that the observed increases in M2 macrophage accumulation in iWAT might largely result from increased M2 cell proliferation.

Another key aspect of our findings is that this cold-stimulated CCL22/CCR4 pathway is dependent on local LNs. This suggests that while M2 macrophages play an important role in inducing CCL22 production, the presence of LNs is necessary to activate the downstream events in the CCL22/CCR4 pathway, leading to increased eosinophil accumulation and iWAT beiging. LNs are known to be both anatomically and functionally associated with WAT health in both rodents and humans (*73*, *74*). Adipocytes residing close to LNs are suggested to be more active in lipolysis in response to local immune assaults, serving as energy reservoirs for the immediate and effective immune response in LNs (*75*–*78*). In line with our research, a recent study also underscores the vital role of LNs in iWAT beiging. They found that cold exposure increases SNS activity within LNs, leading to the secretion of IL-33 from fibroblastic reticular cells into adjacent iWAT (*33*). It remains to be tested whether IL-33, released from fibroblastic reticular cells in LNs, could activate the CCL22/CCR4 axis in iWAT. In support of this model, we observed a decrease in IL-33 levels in iWAT following LNR. IL-33 is known to activate the NF-κB pathway, an essential signaling pathway that regulates immune and inflammatory responses (*79*, *80*). Notably, NF-κB activation has been shown to stimulate CCL22 transcription in murine DCs and B cells (*81*). Therefore, it is reasonable to hypothesize that IL-33 might also activate the NF-κB pathway in macrophages, resulting in increased transcription of CCL22. Unlike IL-33 secretion that is controlled by the SNS, our data suggest that the release of CCL22 by M2 macrophages is not SNS-dependent, indicating other unidentified nonneuronal regulation of the CCL22 regulation. Given the complex interaction between immune signaling in LNs and the regulation of adipose tissue remodeling in response to cold, the specific mechanisms underlying CCL22 up-regulation following LN activation remain an area for further investigation.

In our rescue experiments, we noticed that in the presence of LN, CCL22 injection but not M2 macrophage injection further promoted iWAT beiging. It is plausible that LN not only acts to foster CCL22 induction for iWAT beiging but also controls proper secretion of CCL22 from M2 macrophages for the appropriate amount of beiging. Another potential explanation could be similar to what was found in the macrophage field (*37*), in which there are discrete subpopulations of macrophages that both positively and negatively mediate thermogenesis. Therefore, it could be that LN plays a crucial role in regulating the balance between the positive and negative regulators of thermogenesis, ensuring the proper amount of beiging in response to cold exposure.

Therapeutically, given the ability of CCL22 to induce beige adipocyte thermogenesis and reduce obesity, its therapeutic potential in metabolic diseases is feasible and should be further explored. The effect of an rCCL22 protein used in this study illustrates this potential. We demonstrate that directly activating CCL22 can activate a thermogenic gene program in response to cold exposure, increase energy expenditure, and prevent weight gain upon diet challenge. Conversely, the neutralization of CCL22 by an antibody can block the cold-induced thermogenic gene program. Our observational data in humans are consistent with our mouse data. Circulating CCL22 negatively correlates with adiposity, and weight loss interventions increase serum CCL22, suggesting serum CCL22 as a potential biomarker of adiposity. Although these data provide important proof of concept that therapeutically activating CCL22 could be effective and feasible, we are well aware that CCL22 is elevated in the tumor tissues, which is linked to T_reg_ recruitment and contributes to an immunosuppressive environment for tumors (*82*–*84*). Therefore, other proteins with no cancer-promoting properties would be better options. Thus, a better understanding of the regulation of CCL22 may reveal other pharmacological modulators of circulating CCL22 that can be used to control energy metabolism and reduce adiposity. Considering the important function of eosinophils and M2 macrophages in other repairing activities, including those in impaired muscle (*85*, *86*) and liver (*87*), future investigation is needed for additional potential therapeutic applications.

In summary, we have identified that beige adipogenesis around the inguinal LNs of WAT depends on the CCL22-CCR4 signaling. Our work establishes previously unrecognized biological pathways, including MDC CCL22, its receptor CCR4 in eosinophils, and the upstream influence of LNs in the differentiation of APCs into beige adipocytes, as well as their role in maintaining whole-body energy balance and obesity prevention.

## MATERIALS AND METHODS

### Animal model

All studies were performed according to procedures reviewed and approved by the Institutional Animal Care and Use Committee of University of Illinois at Chicago. Mice were housed in a temperature/humidity-controlled environment (23° ± 3°C/70 ± 10%), and a 14:10 light:dark cycle with a standard rodent chow diet and water unless otherwise indicated. B6J.129(Cg)-Igs2^tm1.1(CAG-Cas9*)Mmw^/JH11^Cas9^ CRISPR-Cas9 knock-in mice were purchased from the Jackson Laboratory (#028239). CCR4 KO mice (spCas9 mice were injected with AAV8-CCR4-sgRNA into biolateral inguinal adipose tissue) were generated on a C57BL/6 background. They were used to investigate the effects of CCL22/CCR4 on beige adipocyte formation. For fate mapping, the R26^RFP^ reporter line was purchased from JAX (#007914, Rosa-CAG-LSL-tdTomato). Sma^Cre-ERT2^ mice were provided by P. Chambon. Pdgfrβ^Cre-ERT2^ mice were provided by H. C. Grajal (available at JAX, #030201). S. Morrison and B. Richardson provided the Pdgfrα^Cre-ERT2^ mice.

### Tamoxifen treatment

To induce RFP expression in vivo, mice were intraperitoneally injected with 50 mg/kg body weight of TAM (Cayman Chemical) dissolved in sunflower oil (Sigma-Aldrich) for two consecutive days as previously described (*28*, *88*, *89*).

### Inguinal LNR protocol

Ten-week-old male C57BL/6 mice were randomly divided into two groups according to body weight (sham or LNR group). After the animal lost righting reflexes by toe pinch reflex assessment using an anesthetic (isoflurane, MO 64068, pivotal), the animal was placed on its side, and an incision area within iWAT was localized and applied by chlorhexidine. A tiny incision (approximately 5 mm) above the hind paw near the abdomen was made with sharp scissors after removing loose hairs with hair removal cream. The LN can now be visualized as grayish as or darker than the surrounding fat by stretching the incision with two forceps. After successfully removing the LN, the fat pads were placed back in their original location, and the skin was closed with 5/0 monofilament nonabsorbable synthetic sutures. The suture was removed 7 to 10 days after surgery.

### Sympathetic denervation of lymph node and inguinal adipose tissue

Ten-week-old male C57BL/6 mice were bilaterally injected 6-OHDA (6-hydroxydopamine, 10 mg/ml) (Cayman Chemicals, 25330) directly into the LN and iWAT. A volume of 10 μl of 6-OHDA was administered into the LN and iWAT using a microsyringe, after which the surgical wounds were closed and secured.

### Isolation and culture of SVF cells

SVF cells were isolated as previously described (*28*). Briefly, following a 7-day cold (6°C) acclimation, subcutaneous iWAT was dissected and digested at 37°C for 40 min in isolation buffer [100 mM (Hepes), 0.12 M NaCl, 50 mM KCl, 5 mM d-glucose, 1.5% bovine serum albumin (BSA), and 1 mM CaCl_2_ (pH 7.3)] containing collagenase type I (1 mg/ml; Worthington Biochemical, LS004197). After removing the floating layer containing MAs, the aqueous phase was centrifuged at 1000*g* for 10 min. The pellet, including a crude SVF, was resuspended in red blood cell lysis buffer (155 mM NH_4_Cl, 10 mM KHCO_3_, and 0.1 mM EDTA) for 5 min and centrifuged at 1000*g* for 5 min. The pellet was washed once with 1× phosphate-buffered saline (PBS), resuspended, and strained through the 70-mm mesh.

The isolated cells were cultured in Dulbecco's modified Eagle's medium (DMEM)/F12 media (Sigma-Aldrich, St. Louis, MO) supplemented with 10% fetal bovine serum (FBS) (Sigma-Aldrich, St. Louis, MO) and 1% penicillin/streptomycin (Gibco, Waltham, MA). Beige adipocytes were induced by treating confluent cells with DMEM/F12 containing 10% FBS, insulin (1 mg/ml; Sigma-Aldrich, St. Louis, MO), 1 mM dexamethasone (Cayman, Ann Arbor, MI), 0.5 mM isobutylmethylxanthine (Sigma-Aldrich, St. Louis, MO), 60 mM indomethacin (Sigma-Aldrich, St. Louis, MO), 1 nM triiodo-l-thyronine (Sigma-Aldrich, St. Louis, MO), and 1 mM rosiglitazone (Sigma-Aldrich, St. Louis, MO) for the first 2 days and with DMEM/F12 containing 10% FBS and insulin (1 mg/ml), 1 nM triiodo-l-thyronine, and 1 mM rosiglitazone every 2 days. All cells were cultured and maintained at 37°C in a 5% CO_2_ incubator unless otherwise indicated.

### Isolation and culture of BMDMs

Bone marrow cells were isolated from the femur and tibia of 10-week-old male C57BL/6 mice and differentiated into mature macrophages for 7 days as described (*41*). Briefly, cells were maintained in DMEM containing 10% FBS and recombinant murine M-CSF (10 ng/ml; PeproTech, 315-02). On day 7, lipopolysaccharide (100 ng/ml; Sigma-Aldrich, L6529) or recombinant murine IL-4 (10 ng/ml; PeproTech, 214-14) was added for M1 or M2 polarization for 4 days, respectively.

### Isolation and culture of mouse BMDEs

Eosinophils were isolated and differentiated from bone marrow cells as described (*90*). Briefly, bone marrow cells were collected from the femurs and tibiae of 10-week-old male C57BL/6 mice and cultured at 10^6^/ml in medium containing DMEM (1×, containing 25 mM Hepes) with 20% FBS (Cambrex), penicillin (100 IU/ml), streptomycin (10 μg/ml), 2 mM glutamine, 1× nonessential amino acids, 1 mM sodium pyruvate, recombinant murine M-CSF (100 ng/ml; PeproTech), and recombinant murine FLT3 ligand (FLT3-L; 100 ng/ml; PeproTech, 250-31L) from days 0 to 4. On day 4, the medium containing stem cell factor and FLT3-L was replaced with a medium containing only recombinant murine IL-5 protein (10 ng/ml; Novus Biological, NBP2-35132). On day 8, the cells were moved to a fresh medium supplemented with IL-5 (10 ng/ml). Every other day, one-half of the medium was replaced with fresh medium containing IL-5 (10 ng/ml).

### Cold exposure in vivo

For cold exposure experiments, mice were placed in a 6°C environmental chamber (Environmental & Temperature Solutions) for 7 days. Body temperature was measured using a rectal probe (Physitemp Instruments Inc.). The probe was lubricated with glycerol and inserted 1.27 cm (0.5 inch), and the temperature was recorded when stabilized at the indicated time points.

### Cold exposure assay in vitro

To mimic the conditions of cold exposure in vivo, mice were pre-exposed to a temperature of 6°C for 7 days, and then the SVF cells were isolated and cultured at 31°C, a temperature known to induce the expression of UCP1 in isolated cells (*50*). This temperature (31°C) does not interfere with the survival of M2 macrophage cells (*41*). BMDMs or BMDEs were cultured and differentiated in a 37°C incubator and then cocultured with SVF in a 31°C incubator for further studies.

### In vitro coculture assay

M2 macrophages were collected from BMDMs and polarized by IL-4 (10 ng/ml). To investigate the effect of M2 macrophages on LN removal in mouse adipocytes, M2 macrophages were cultured with SVF from iWAT by using 24-well transwell inserts (Corning, GLS3464). For the coculture assay, an 8-μm polycarbonate membrane insert was used in the coculture system, where M2 macrophages were in the upper compartment and SVF cells were in the lower compartment.

### M2 macrophage transfer assay

Briefly, M2 macrophages were collected from BMDM-derived and polarized by IL-4 (10 ng/ml), according to a standard procedure (*41*). The same number (about 2 × 10^6^) of LN removal reduced M2 macrophages were injected into the bilateral iWAT every 2 days for a total of three times. The mice were housed in a 6°C chamber for a total of 7 days.

To test the survival rate of injected M2 macrophages in iWAT, we genetically labeled the exogenous M2 macrophages with RFP. Briefly, we used the “germline Cre” leaky mice from the original breeding (Tre-Cre; Rosa26-RFP). Tre-Cre was unexpectedly leaky in the germline, which induced recombination and RFP expression in all the cells and passed to its future generations. Therefore, the RFP was driven by “ubiquitous”-Cre, and labeled all the cells from the body, including the BMDM-derived M2 macrophages. We injected RFP-labeled M2 macrophages into iWAT, and the count of these RFP-M2 macrophages was then measured on days 1 and 2 after injection.

### Flow cytometry and sorting

Flow cytometry was performed using a BD LSRFortessa and BD LSRFortessa X-20. Sorting by FACS was performed using a BD Influx and BD FACSAria II. SVF cells were isolated from 10-week-old male mice. Cells were washed with PBS and incubated with fluorochrome-conjugated antibodies against surface antigens. Stained cells were analyzed with a BD FACS verse flow cytometer. All cells were pre-blocked with anti-CD16/32 (BioLegend, #101302) Fc block to reduce nonspecific binding, and UltraComp eBeads (Invitrogen) were used for single-stained compensation controls. For all sorted cells, we conducted post-sorting verifications by reanalyzing the catch tube to confirm the cells were within the designated sorting gate.

To identify M2 macrophages, cells were stained with a combination of the following antibodies: CD45 (PerCP, BioLegend, #103132), F4/80 [fluorescein isothiocyanate (FITC), BioLegend, #123107], CD206 [phycoerythrin (PE), BioLegend, 141705], and CD64 (BV605, BioLegend, #139323). M2 macrophages were defined as live CD64^+^CD45^+^F4/80^+^CD206^+^ cells.

To identify eosinophils, cells were stained with a combination of the following antibodies: CD45 (PerCP, BioLegend, #103132), F4/80 (FITC, BioLegend, #123107), CD11b (BV605, BioLegend, #101257), and SiglecF/CD170(APC, BioLegend, #155507). Eosinophils were defined as live CD45^+^ CD11b^+^ F4/80^+^ SiglecF^+^ SSChi cells.

To identify T_reg_ cells, cells were stained with a combination of the following antibodies: CD4 (PerCP/Cyanine 5.5, BioLegend, #100433), CD25 (APC, BioLegend, #102011), CD127 (FITC, BioLegend, #158207), and FoxP3(PE, BioLegend, #126403). T_reg_ cells were defined as live CD4^+^ CD25^+^ CD127^+^ FoxP3^+^ cells.

To identify DCs, cells were stained with a combination of the following antibodies: CD11c (FITC, BioLegend, #126403), MHCII (PerCP, BioLegend, #107623), and CD103 (PE, BioLegend, #156903). DCs were defined as live CD11c^+^ MHCII^+^ CD103^+^ cells.

To identify T_H_2 cells, cells were stained with a combination of the following antibodies: T1/ST2 (FITC, Morwell MD Biosciences, NC9973646), Gata-3 (eFluor 660, eBioscience, 50-9966-42), CD45 (PerCP, BioLegend, #103132), FoxP3 (PE, BioLegend, #126403), CD4 (BV650, BioLegend, #100469). T_H_2 cells were defined as live CD45^+^ CD4^+^ FOXP3^−^ GATA3^+^ T1/ST2^+^ cells.

For RFP^+^ sorting, live SVF cells from TAM-injected Sma-RFP, Pdgfrα-RFP, and Pdgfrβ-RFP mice were sorted on the basis of their native fluorescence. To determine background fluorescence levels, SVF cells from mice not injected with TAM were used.

For delayed flow cytometric analysis, fully stained cells were fixed with 1% paraformaldehyde. In these instances, ZombieNIR (BioLegend), a fixable viability dye, was used to identify live cells. Flow cytometry analysis was performed using FlowJo software v10.

### Inhibitor assay in vitro

SVF cells or BMDE (eosinophils) from 10-week-old male C57BL/6 mice were cultured in 24-well plates. After the cells were stable, SVF cells were treated with vehicle or AZD2098 (CCR4 antagonist, 100 nM) (Ambeed Inc., A924249), vehicle or dupilumab (IL-4Rα inhibitor, 50 μM) (Selleck Chemicals, A2038), and vehicle or AS1517499 (p-STAT6 inhibitor, 50 μM) (Cayman Chemical, 29071). Eosinophils were treated with vehicle or PF573228 (FAK inhibitor, 5 μM) (Sigma-Aldrich, PZ0117). Then, CCL22 (10 ng/ml) was added to investigate the function of the CCR4 or p-FAK/IL-4 pathway in CCL22-induced beige adipocyte formation.

### RNA interference in M2 macrophages

RNA interference was conducted as previously described (*91*). Briefly, the CCL22 siRNA and NC siRNA were purchased from Sigma-Aldrich and transfected into BMDMs by using Lipofectamine reagents (Invitrogen, Carlsbad, CA, USA) according to the manufacturer’s instructions. The sequence of siRNA targeting CCL22 is 5′- CAGCCUUACCCAAUGCCUA -3′ (sense) and 5′- UAGGCAUUGGGUAAGGCUG -3′ (antisense). The siRNA targeting control was purchased from Santa Cruz Biotechnology lnc. (SC37007).

### Cell viability test

Cell viability was tested by 3-(4,5-dimethylthiazol-2-yl)-2,5-diphenyltetrazolium bromide (MTT) assay. MTT (5 mg/ml) was added to MTT solution to each well at a final concentration of 0.5 mg/ml. Incubate the cells with MTT solution for 4 hours at 37°C in a tissue culture incubator. After the incubation period, we carefully remove the MTT solution from each well without disturbing the formazan crystals. We add a 100 μl of DMSO to each well. We incubate the plate for about 30 min at RT to allow for complete dissolution of the formazan crystals. We use a plate reader to measure the absorbance at 570 nm.

### Histological staining

H&E staining was conducted on paraffin sections using standard methods as described previously (*28*). Briefly, adipose tissues were fixed in formalin overnight, dehydrated, embedded in paraffin, and sectioned with a microtome at 5 μm thicknesses. For IF staining, paraffin sections were incubated with permeabilization buffer (0.3% Triton X-100 in PBS) for 30 min at RT (23°C), with primary antibody at 4°C overnight, and with secondary antibody for 2 hours at RT. Antibodies used for immunostaining were rabbit anti-UCP1 (1:500; Abcam, ab23841), mouse anti-IB4 (1:500; biotinylated, B-1205-5), goat anti-perilipin (1:500; Abcam, ab61682), rabbit anti-CCR4 (1:200; Novus Biological, NB56336SS), mouse anti-CD206 antibody (Bio-Rad, MAC2235GA), rat anti-F4/80 (1:500; ab6640, Abcam), rabbit anti-p65 (1:1000; Abcam, ab32536), 4′,6-diamidino-2-phenylindole (DAPI; Vector Laboratories, SP-8500). Secondary antibodies including cy3 donkey anti-mouse, Alexa donkey anti-rabbit, cy5 donkey anti-rat, and cy5 donkey anti-goat were purchased from Jackson ImmunoResearch. All secondary antibodies were used at a 1:500 dilution. Immunostaining images were taken using a Leica DMi8 microscope (Leica, Wetzlar, Germany) or a confocal microscope (LSM880).

### Western blotting analysis

Western blotting analysis was performed as described previously (*92*). Total protein lysates (20 μg) were immunoblotted with rabbit anti–p-FAK (Y397) (1:1000; ABclonal, #AP0302), rabbit anti-FAK (1:1000; ABclonal, A11195), rabbit anti-p65 antibody (1:1000; Abcam, ab32536), rabbit anti-CCR4 antibody (1:1000; Novus Biological, NB56336SS), rabbit anti-UCP1 antibody (1:500; Abcam, ab23841), rabbit anti-tubulin antibody [1:2000; Cell Signaling Technology (CST), 2146S], mouse anti-CCL22 antibody (1:1000; R&D Systems, MAB439-SP), mouse anti-CD206 antibody (Bio-Rad, MAC2235GA), rat anti-F4/80 (1:500; Abcam, ab6640), rat anti-siglecF (1:500; Novus Biological, NBP1-91149), rabbit anti–IL-13 antibody (1:500; ABclonal, A2089), rabbit anti-p-STAT6 antibody (1:500; ABclonal, AP0456), rabbit anti-STAT6 antibody (1:500; ABclonal, A0755), rabbit anti-Histone3 antibody (1:1000; CST, 4499P), followed by goat anti-rat horseradish peroxidase (HRP)–conjugated secondary antibody (1:5000; ABconal, AS028), anti-rabbit HRP-conjugated secondary antibody (1:5000; CST, 7074S), goat anti-mouse HRP-conjugated secondary antibody (1:5000; CST, 96714S). The levels of tubulin served as the loading control.

### Quantitative real-time PCR

qPCR assays were conducted as described previously (*28*). Total RNA was extracted using TriPure Isolation Reagent (Roche, Basel, Switzerland) from adipose tissues using Bullet Blender Homogenizer (Next Advance, Troy, NY, USA) according to the manufacturer’s protocol. cDNA was generated from 1 μg of total RNA using a High-Capacity cDNA Synthesis kit (Bio-Rad Laboratories, Hercules, CA, USA), and qPCR was performed using 2× Universal SYBR Green Fast qPCR Mix (Abclonal) following the manufacturer’s instructions and analyzed with a ViiA7 system (Applied Biosystems, Foster City, CA, USA). Data were analyzed using the comparative Ct method, and mRNAs’ relative expression was determined after normalization to *Actin*. Primer sequences are available in Table 1.

**Table 1.** Primer sequences for qPCR gene expression analysis.

Species	Gene abbreviation	Forward primer (5′-3′)	Reverse primer (5′-3′)
Mouse	*Ucp1*	ACTGCCACACCTCCAGTCATT	CTTTGCCTCACTCAGGATTGG
	*Tmem26*	CGGCCATCTTTGTGTACCTG	TGTGGGATGACAGGGTTTGA
	*Cd137*	CGTGCAGAACTCCTGTGATAAC	GTCCACCTATGCTGGAGAAGG
	*Tnf-α*	CCTGTAGCCCAGGTCGTAG	GGGAGTAGACAAGGTACAACCC
	*Tnf-β*	CTCCCGTGGCTTCTAGTGC	GCCTTAGTTTGGACAGGATCTG
	*Arg1*	CTCCAAGCCAAAGTCCTTAGAG	AGGAGCTGTCATTAGGGACATC
	*Fizz1*	CCCTCCACTGTAACGAAGACTC	CACACCCAGTAGCAGTCATCC
	*iNOS*	GTTCTCAGCCCAACAATACAAGA	GTGGACGGGTCGATGTCAC
	*Siglecf*	CAGGGACGTACTTCTTCAGATTGG	GGGTAGATGTGACTTGGATGTTAGG
	*Il1α*	GCAACGGGAAGATTCTGAAG	TGACAAACTTCTGCCTGACG
	*Il2*	CCT GAG CAG GAT GGA GAA TTA CA	CGC AGA GGT CCA AGT TCA TCT
	*Il3*	CCT GGG ACT CCA AGC TTC AA	GAC AAT AGA GCT GCA ATT CAA CGT
	*Il4*	GACGCCATGCACGGAGAT	GCCCTACAGACGAGCTCACTCT
	*Il5*	TCAGACTGTGCCATGACTGT	AGAAGTAAGGCCCAGCATGT
	*Il6*	CCTCTCTGCAAGAGACTTCCAT	AGTCTCCTCTCCGGACTTGT
	*Il10*	GCTCTTACTGACTGGCATGAG	CGCAGCTCTAGGAGCATGTG
	*Il11*	TGAACTGTGTTTGTCGCCTG	TCTGAAGAGACTCGAGGGGA
	*Il12*	CTCACCTGTGACACGCCTGA	CAGGACACTGAATACTTCTC
	*Il13*	TGGTTCTCTCACTGGCTCTG	GGGAGTCTGGTCTTGTGTGA
	*Il16*	CAGCCATTCAGCCTACACCA	CGTCCTCCATCTTGCTTTCC
	*Il25*	ATGTACCAGGCTGTTGCATTCTTG	CTAAGCCATGACCCGGGGCC
	*Ccl3*	TTCTGCTGACAAGCTCACCCT	ATGGCGCTGAGAAGACTTGGT
	*Ccl5*	AGATCTCTGCAGCTGCCCTCA	GGAGCACTTGCTGCTGGTGTAG
	*Ccl11*	CATGACCAGTAAGAAGATCCC	CTTGAAGACTATGGCTTTCAGG
	*Ccl17*	GCTGCTGTCCATGGTTTCAA	TTTGTGTTCGCCTGTAGTGC
	*Ccl20*	GAACTGGGTGAAAAGGGCTG	TCAACCCCAGCTGTGATCAT
	*Ccl22*	ATATCTGTGCCGATCCCAGG	GGCAGAAGAATAGGGCTTGC
	*Ccl24*	CGGCCTCCTTCTCCTGGTA	TGGCCAACTGGTAGCTAACCA
	*Cxcl1*	CTGCACCCAAACCGAAGTC	AGCTTCAGGGTCAAGGCAAG
	*Cxcl5*	AATCTCCACACCTCCTCCAG	AGCGTGAACAGCAACAGAAA
	*Cxcl9*	CTGGGGTTAAAGGTGTGTGC	CTCTGTGCGCTGAAGATGTC
	*Cxcl10*	GCCGTCATTTTCTGCCTCAT	GATAGGCTCGCAGGGATGAT
	*Cxcl11*	AGGAAGGTCACAGCCATAGC	CGATCTCTGCCATTTTGACG
	*β-Actin*	CCACTGGCATCGTGATGGACTCC	GCCGTGGTGGTGAAGCTGTAGC
Human	*Ucp1*	AGAAGGGCGGATGAAACTCT	ATCCTGGACCGTGTCGTAG
	*Tmem26*	TGCTGCAGTTTCCACTTGAC	GATGTTCCACAGATCGGCAC
	*Cd137*	GTAACACGACATGCTCCACC	GGACAAAGGCAGAAGGTGTG
	β-*Actin*	GTAGTTTCGTGGATGCCACAG	GAGCTACGAGCTGCCTGACG

### Transcriptomics

At 10 weeks of age, male C57BL/6 mice received sham or LN removal surgery. After recovery at RT (23°C) for 10 days, mice were housed under cold exposure (6°C) for 7 days with a chow diet. Then, RNA was extracted from iWAT and sent for sequencing (LNs were removed from the sham and LNR groups when RNA was extracted). Quantified libraries will be pooled and sequenced on Illumina platforms, according to effective library concentration and data amount. The clustering of the index-coded samples was performed according to the manufacturer’s instructions. After cluster generation, the library preparations were sequenced on an Illumina platform, and paired-end reads were generated. GO enrichment analysis of differentially expressed genes was implemented by the clusterProfiler R package, in which gene length bias was corrected. GO terms with corrected *P* value less than 0.05 were considered significantly enriched by differentially expressed genes. Genes with log_2_FC ≥ 1 and − log_10_*P* value ≥ 1.3 were considered significant. Our RNA-seq data in GEO data in NCBI: GSE245718.

### Serum or cell CCL22 concentration measurement

Serum or cell CCL22 concentrations were measured using a CCL22/MDC PicoKine ELISA kit (Boster Bio, EK0447). For the serum CCL22 concentration test, blood from humans and mice was centrifuged for 20 min at 4°C at 12,000*g*, and then serum was used to test CCL22 levels. For the cell CCL22 concentration test, the cells were collected and centrifuged for 20 min at 4°C at 12,000*g*, and then the supernatants were used to test CCL22 levels. The CCL22 level was normalized to the total protein concentration of the cells.

### Nuclear and cytoplasmic protein extraction and NF-κB (p65) translocation

Nuclear and cytoplasmic proteins were extracted by a manufacturer kit (AR0106, Boster Bio). Eosinophil cells were cultured in 24-well plates with an adhesive coverslip. After approximately 50% coverage of the coverslip, the cells were treated with vehicle or CCL22 (10 ng/ml) for 48 hours. CCR4 was inhibited as described above. FAK was inhibited by the FAK inhibitor PF573228. The FAK signaling pathway was further tested by Western blotting. Cell-climbing slices were rinsed three times in PBS, fixed in paraformaldehyde for 10 min, and washed in 0.4% Triton X-100 (T9284; Sigma-Aldrich) for 30 min. After 1 hour of blocking in 3% goat serum at RT, the slices were incubated overnight in rabbit anti–NF-κB (p65) (1:1000) at RT. The next day, the slices were rinsed three times in PBS and incubated in goat anti-rabbit FITC-conjugated secondary antibody. Then, the slices were covered with a DAPI mounting medium. Fluorescence images were obtained using a confocal microscope.

### Metabolic phenotyping studies

To investigate the metabolic effects of CCL22 on mice fed chow, male C57BL/6 mice at 10 weeks of age were randomly assigned to be injected into iWAT with saline or rCCL22 (20 μg/kg per day, PeproTech, 250-23) every other day for a total of 2 weeks and housed at 6°C. Body weight and food intake were monitored every other day, and core temperature (Thermalert Model TH-8 Temperature Monitor, Physitemp Instruments Inc., USA) and blood glucose (Contour, USA) were monitored every week. At the end of the experiment, mice were deeply anesthetized and euthanized. Tissues were isolated and weighed. An aliquot of iWAT was collected for H&E staining and IF staining. iWAT was used to determine the mRNA and protein expression of *Ucp1*, *Tmem26*, and *Cd137*.

To investigate the metabolic effects of CCL22 on DIO mice, male C57BL/6 mice at 10 weeks of age fed an HFD were randomly assigned to be injected into iWAT with saline or rCCL22 every other day for a total of 2 weeks and housed at 6°C. At the end of the experiment, body composition was determined using a nuclear magnetic resonance system (Body Composition NMR Analyzer, LF50, the minispec, Bruker). Core temperature was measured. Mice were then housed individually and acclimatized to the metabolic chambers (Promethion Metabolic Screening Systems, USA) at the UIC Biologic Resources Laboratory for 2 days before data collection was initiated. For the subsequent 3 days, food intake, VO_2_, VCO_2_, RER, and locomotive activity were monitored over a 12-hour light/dark cycle with food provided ad libitum. For energy expenditure analysis, embedded analysis of covariance (ANCOVA) tools in a web application CalR (*93*) were used to perform regression-based indirect calorimetric analysis. Subsequently, tissues were isolated and weighed. An aliquot of iWAT was collected for H&E staining and IF staining. iWAT was used to determine the mRNA and protein expression of *Ucp1*, *Tmem26*, and *Cd137*. Serum was collected and used for CCL22 level measurement.

### GTT and insulin tolerance test

GTT and insulin tolerance test (ITT) were performed as described before (*94*). Briefly, intraperitoneal GTT was performed after overnight fasting. For GTT, an injection of glucose (1 g/kg body weight) was given to the mice, and blood glucose levels were measured subsequently at 0, 20, 40, 60, 80, 100, and 120 min. ITT was performed after 4 hours of fasting. The mice were intraperitoneally injected with a single dose of insulin (1 U/kg body weight), after which the blood glucose levels were measured at 0, 20, 40, 60, 80, 100, and 120 min. At 10 weeks of age, C57BL/6 male mice were fed with HFD and injected into iWAT with vehicle or rCCL22 (20 μg/kg per day) every other day for 2 weeks, and then GTT was performed. Another group of mice received the same treatment for 2 weeks and then underwent ITT.

### OCR analysis

Cellular OCR was determined using an Agilent Seahorse XFp Analyzer (Agilent Technologies, S7802A) as described previously (*92*). SVF from 10-week-old male C57BL/6 mouse iWAT was plated and differentiated in Seahorse XFp Cell Culture Miniplates (Agilent Technologies, 103022100). The assay medium was prepared by supplementing Agilent Seahorse XF Base Medium (Agilent Technologies, 102353-100, 103193-100, and 103334-100). Agilent Seahorse recommends 1 mM pyruvate (Sigma-Aldrich, S8636), 2 mM glutamine (Sigma-Aldrich, G8540), and 10 mM glucose (Sigma-Aldrich, G8769) as starting points. Before analysis, the adipocyte culture medium was changed to a respiration medium consisting of DMEM lacking NaHCO_3_ (Sigma-Aldrich), NaCl (1.85 g/liter), phenol red (3 mg/liter), 2% fatty acid–free BSA, and sodium pyruvate (1 mM), and the pH was adjusted to 7.4. Basal respiration was determined to be the OCR in the presence of substrate (1 mM sodium pyruvate) alone. The assay medium was warmed to 37°C. The pH was adjusted to 7.4 with 0.1 N NaOH. Oligomycin inhibits adenosine 5′-triphosphate (ATP) synthase (complex V), and the decrease in OCR relates to the mitochondrial respiration associated with cellular ATP production after injection of oligomycin (1 μM). Spare respiratory capacity, defined as the difference between maximal and basal respiration, can be calculated by the carbonyl cyanide-4-(trifluoromethoxy) phenylhydrazone–stimulated (2 μM) OCR. The combination of complex I inhibitor rotenone (0.5 μM) and complex III inhibitor antimycin A (0.5 μM) can shut down mitochondrial respiration and enable the calculation of nonmitochondrial respiration driven by processes outside the mitochondria. The Agilent Seahorse XF Cell Mito Stress Test Report Generator automatically calculates the Agilent Seahorse XFp Cell Mito Stress Test parameters from Wave Data.

### CCR4 KO mouse model

B6J.129(Cg)-Igs2^tm1.1(CAG-Cas9*)Mmw^/JH11^Cas9^ CRISPR-Cas9 knock-in mice were purchased from the Jackson Laboratory (#028239). These Cas9 mice constitutively express CRISPR-associated protein 9 (Cas9) endonuclease directed by a CAG promoter. An AAV virus vector carrying paired sgRNAs (AAV-sgRNAs-CCR4) targeting the CCR4 gene was generated and packaged by Vector Builder (USA). The CCR4 target sgRNA sequences were as follows: sgRNA-A1: TGTACGGCGTCCAGAAGCCG, sgRNA-A2: TCGTCGAGTTGACCGAGTAC. For 8-week-old mice, male Cas9 mice were injected into iWAT with AAV-sgRNAs-CCR4 (2 × 10^13^ genome copies/ml) to generate a CCR4 deletion mouse model (CCR4^−/−^). Bilateral iWAT was injected with 20 μl of virus in three positions (anterior, middle, and posterior), respectively. After 2 weeks, this mouse model was used to study the function of CCL22/CCR4 in beige adipocyte formation.

### Human participants

Plasma samples used in this trial were from three previously published human weight loss trials (*62*–*64*). Briefly, participants participated in a dietary weight loss intervention for 2 to 6 months. Participants were recruited from the Chicago area using flyers placed around the University and screened via a questionnaire, BMI assessment, and pregnancy test. Inclusion criteria: female, male, age between 18 and 65 years, BMI between 30 and 50 kg/m^2^, and previously sedentary (<60 min/week of light activity for the 3 months before the study). Exclusion criteria: history of diabetes mellitus, use of medications that could affect body weight, weight unstable for 3 months before the beginning of the study (>4 kg weight loss or gain), perimenopausal or otherwise irregular menstrual cycle, nightshift workers, pregnant or trying to become pregnant, and current smokers. Body weight was measured in a hospital gown with a digital scale. Fat mass, lean mass, visceral fat mass, and bone density were measured in the fasted state by dual-energy x-ray absorptiometry (iDXA, GE HealthCare). Blood samples were obtained following a 12-hour fast at baseline and posttreatment. The protocols were approved by the Office for the Protection of Research Subjects at University of Illinois at Chicago, and informed consent was obtained from all participants. The clinical characteristics of all human subjects used in this study are detailed in Table 2.

**Table 2.** Clinical characteristics of all human participants.

No.	Gender	Age	Weight (M0) (kg)	Weight (M3) (kg)	Weight (M6) (kg)	BMI (M0) (kg/m^2^)	Fat mass (M0) (kg)	Glucose (M0) (mg/dl)
1	\	\	121.6	120.3	120.3	38	50.6	108.0
2	\	\	145.6	142.4	140.9	43	63.6	113.0
3	\	\	118.3	114.7	114.7	43	53.2	105.0
4	\	\	127.5	127.0	127.0	44	66.7	87.0
5	\	\	90.8	84.0	84.0	35	48.7	87.0
6	\	\	89.5	84.4	84.4	41	48.3	100.0
7	\	\	111.5	110.3	109.3	39	52.2	92.0
8	\	\	93.5	91.4	88.9	33	41.1	92.0
9	\	\	77.6	79.5	79.5	35	33.8	85.0
10	\	\	110.4	103.8	103.2	37	46.8	78.0
11	\	\	83.8	81.8	81.8	32	38.4	77.0
12	\	\	94.9	91.2	88.4	30	36.2	88.0
13	\	\	97.5	83.6	69.9	40	51.6	78.0
14	\	\	115.2	105.3	95.1	36	41.3	96.0
15	\	\	101.7	96.0	96.0	33	35.6	102.0
16	\	\	83.6	80.1	80.1	30	28.2	81.0
17	\	\	94.5	92.7	93.7	36	45.6	90.0
18	\	\	94.7	86.5	87.5	34	46.3	105.0
19	\	\	91.4	88.9	88.9	36	44.0	78.0
20	\	\	77.7	74.0	70.8	32	36.1	85.0
21	61	F	69.3	67.36	\	30.0	31.2	\
22	35	F	81.2	72.55	\	30.6	26.7	\
23	60	F	78.5	73.55	\	31.4	31.3	\
24	48	F	75.6	75.55	\	35.0	35.3	\
25	37	F	85.9	85.82	\	31.9	39.0	\
26	40	F	77.9	75.27	\	35.1	32.7	\
27	51	F	85.4	85.00	\	31.7	38.0	\
28	53	F	82.5	73.1	67.7	31.0	34.2	\
29	64	F	79.5	76.7	74.2	34.4	33.0	\
30	56	F	72.5	69	67.4	31.4	26.8	\
31	42	F	88.6	82.7	81.5	34.6	43.2	\
32	58	F	86.7	81.2	83.4	30.0	33.6	\
33	46	F	82.3	77.8	79.4	33.0	40.6	\
34	43	F	88.7	83.7	85.7	35.5	39.4	\
35	56	F	82.5	79.4	85.4	34.3	40.8	\
36	44	F	78.5	77.1	77.3	30.7	34.5	\
37	51	F	72.1	66.4	65.7	31.2	31.0	\
38	58	F	88.1	83.3	85.4	40.8	43.9	\
39	52	F	76.19	72.4	\	34	34.2	\
40	43	F	69.02	69	\	30.2	29	\

### Association between plasma CCL22 level and body weight in humans

A set of 40 human blood samples were collected and stored in EDTA tubes. Plasma samples were obtained by centrifugation (4°C, 4000*g* for 20 min). The trial investigated the relationship between serum CCL22 concentration and weight changes at 0 months and 6 months. The plasma CCL22 levels were measured by a CCL22/MDC PicoKine ELISA kit (EK0447, Boster Bio). For the two-tailed Pearson’s correlation coefficient analysis, negative r values indicate a negative correlation between human plasma CCL22 levels and body weight or fat content.

### Statistics

Statistical analyses were performed using GraphPad Prism 9.0 statistics software (Chicago, IL, USA). Statistical analysis methods were chosen on the basis of the design of each experiment and are indicated in the figure legends. No animals or data were excluded from the analyses. The data are presented as means ± SEM. P ≤ 0.05 was considered statistically significant. No statistical methods were used to pre-determine sample sizes but our sample sizes are similar to those reported in previous publications (*28*, *89*). Data distribution was assumed to be normal, but this was not formally tested; therefore, data distributions are visualized in each figure. Data collection and analysis were not performed blinded to the conditions of the experiments. Animals (*n* = 3 to 5) in the same cage received the same treatment, and experiments were performed on two or three independent cohorts unless specified. All cell culture experiments were randomly assigned to experimental conditions. For microscopic images, the image fields of IF and H&E were randomly acquired. For flow analysis and qPCR experiments, the analyses were performed in two or three independent experiments; no inconsistent results were observed. NIH Fiji Image and ImageJ software were used for co-localization quantification, evaluating three random fields from a minimum of three mice per cohort. Image acquisition and analysis were conducted using the Leica Application Suite X Microscope software. Excel was used for the collection, analysis, and quantification of raw data.

### Study approval

This research complies with all relevant ethical regulations. Animal experiments were conducted in accordance with NIH guidelines and were approved by the Institutional Animal Care and Use Committee of the University of Illinois at Chicago.
