# Supplementary material for: Macrophage-derived chemokine CCL22 establishes local LN-mediated adaptive thermogenesis and energy expenditure
Source: Sci Adv. 2024 Jun 26;10(26):eadn5229. doi: 10.1126/sciadv.adn5229 (PMC11204298; doi:10.1126/sciadv.adn5229)
Supplement: Supplementary file 1 — Figs. S1 to S18 [file sciadv.adn5229_sm.pdf]

Science Advances
AAAS

# Supplementary Materials for

## Macrophage-derived chemokine CCL22 establishes local LN-mediated adaptive thermogenesis and energy expenditure

Yexian Yuan *et al.*

Corresponding author: Yuwei Jiang, yuweij@uic.edu

*Sci. Adv.* **10**, eadn5229 (2024)
DOI: 10.1126/sciadv.adn5229

**This PDF file includes:**

Figs. S1 to S18

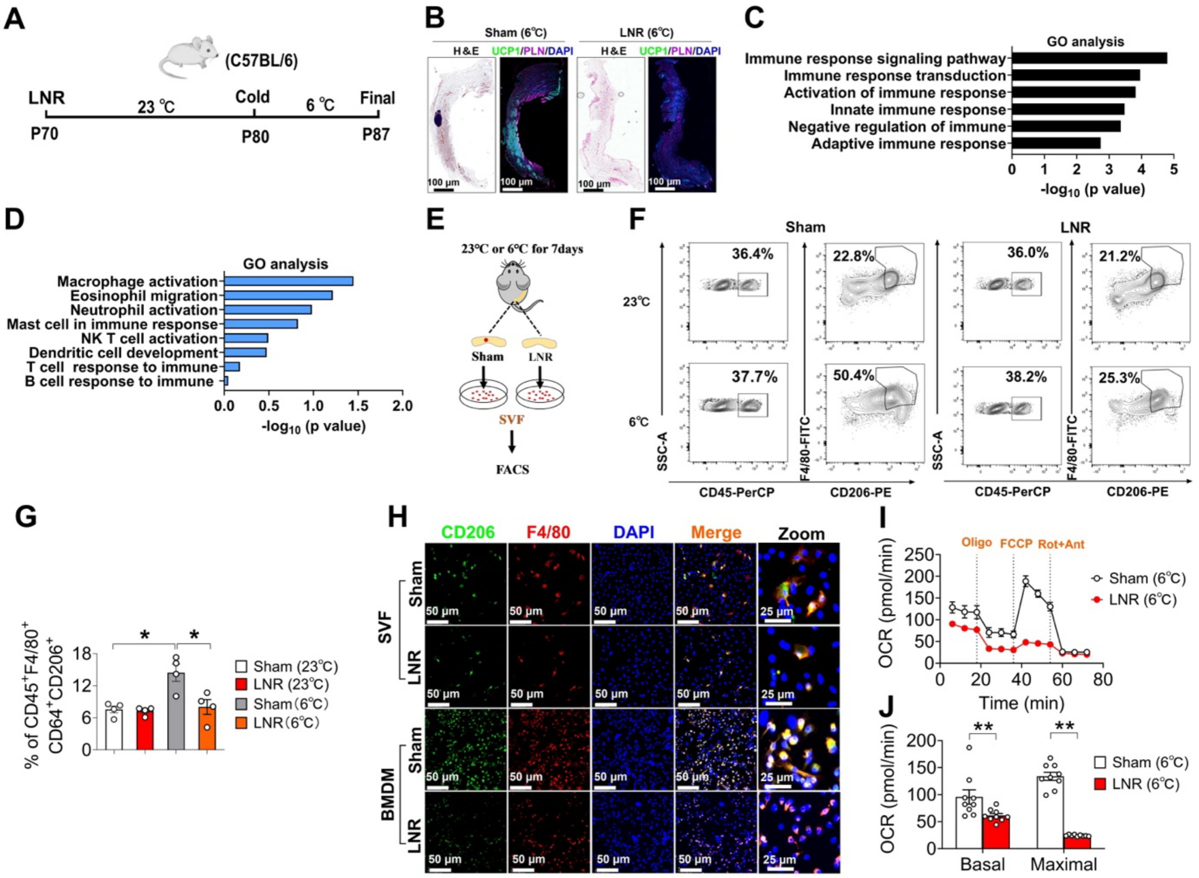


**Fig. S1 (related to Fig. 1): Lymph node removal impairs cold-induced beige adipocyte formation and M2 macrophage accumulation during this process.**

(**A**) Schematic representation of lymph node removal mice. Ten-week-old C57BL/6 male mice received bilateral lymph node (inlaid in iWAT) removal (LNR) or sham in iWAT. After 10 days of recovery at room temperature (23°C), mice were housed at room temperature (23°C) or cold exposure (6°C) for 7 days with a chow diet. (**B**) H&E staining and UCP1 staining of iWAT. Scale bar, 50 μm. (**C**) Gene Ontology (GO) analysis of immune response to LNR. (**D**) GO analysis of innate immune and adaptive immune response to LNR. (**E**) Schematic representation of flow analysis of LNR or sham mice. (**F** and **G**) Flow analysis of M2 macrophages (F4/80$^{hi}$CD45$^{+}$CD64$^{+}$CD206$^{+}$) in iWAT from 10-week-old C57BL/6 male mice housed at 23°C or exposed to 6°C for 7 d with chow feeding (n=4 per group). (**H**) Immunofluorescence of F4/80 and CD206 in iWAT SVF or bone marrow from 10-week-old C57BL/6 male mice exposed to 6°C for 7 d with chow feeding. Scale bar, 50 μm. (**I** and **J**) Oxygen consumption rate in beige adipocytes. SVF cells were isolated from 10-week-old male C57BL/6 mouse iWAT and then induced to differentiate into beige adipocytes for 4 days (n=9 per group). Data information: Results are presented as means ± SEM. In (G, J) *P ≤ 0.05 and **P ≤ 0.01by nonpaired Student's t test.

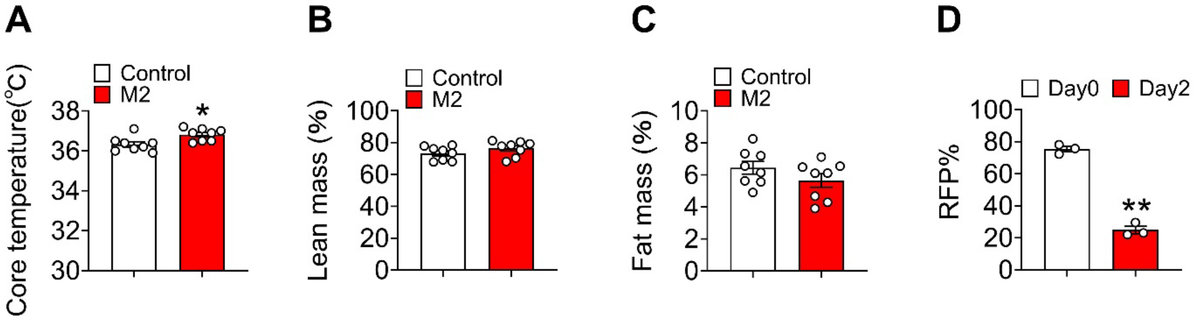


**Fig. S2 (related to Fig. 2): The effects of M2 macrophage injection on mice core temperature, body composition, and the number of M2 macrophages in iWAT post-injection.**

(**A** to **C**) Core temperature (**A**), lean mass (**B**), fat mass (**C**). Ten-week-old C57BL/6 male mice were exposed to 6°C, and M2 macrophages were injected into bilateral iWAT for 7 d with chow feeding (n=8 per group). (**D**) Flow analysis of the number of RFP-labeled M2 macrophages in iWAT (n=3 per group). Mice were injected with RFP-labeled M2 macrophages into iWAT, and the count of these RFP-M2 macrophages was then measured on days 1 and 2 post-injection. Data information: Results are presented as means ± SEM. In (**A** to **D**) *P ≤ 0.05 and **P ≤ 0.01 by nonpaired Student's t test.

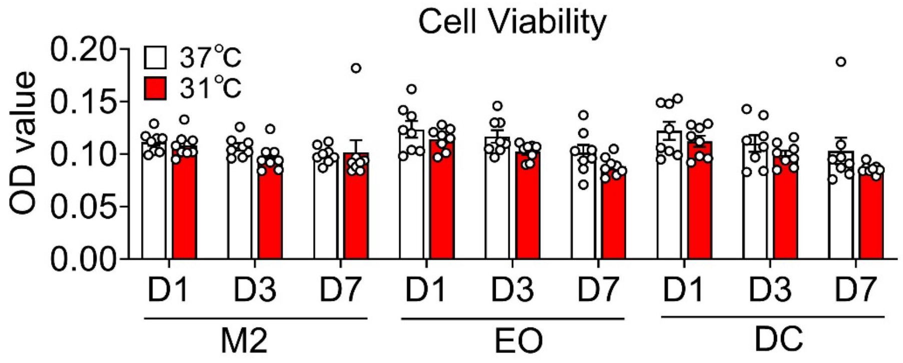


**Fig. S3 (related to Fig. 2): Cell viability between cells incubated at 31°C and those at 37°C.** Cell viability test of M2 macrophages (M2), eosinophils (EO), and dendritic cells (DC) on days 1, 3, and 7 incubated at 31°C and 37°C. Data information: Results are presented as means ± SEM. *P ≤ 0.05 by nonpaired Student's t test.

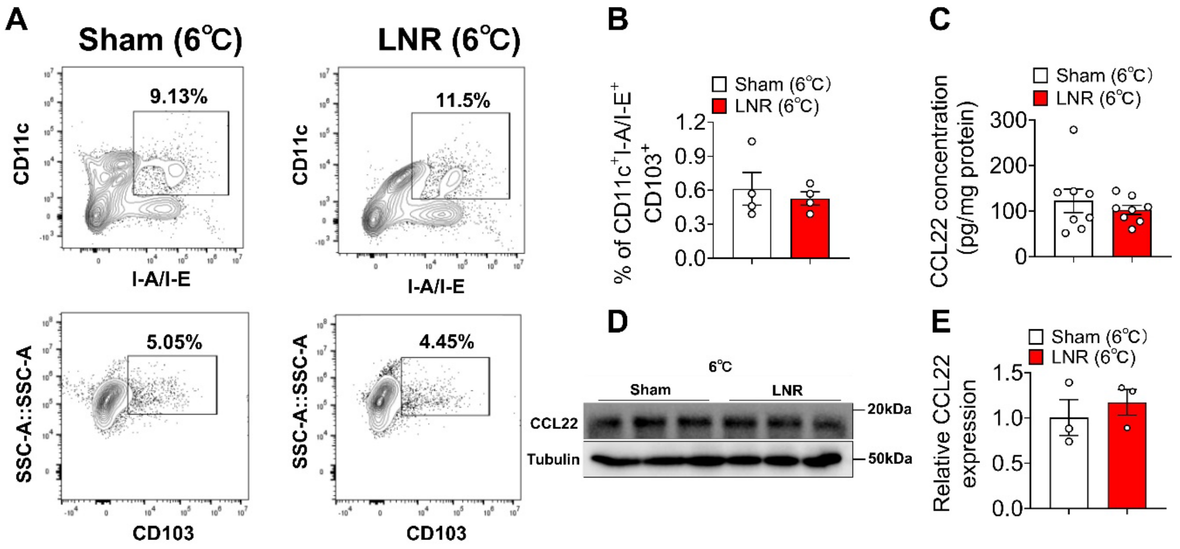


**Fig. S4 (related to Fig. 3): Lymph node removal does not affect CCL22 secretion from dendritic cells.**

(**A** and **B**) Flow cytometry analysis of dendritic cells ($CD11c^{+}I\text{-}A/I\text{-}E^{+}CD103^{+}$) in iWAT. Ten-week-old C57BL/6 male mice received LNR or sham in iWAT. After 10 days of recovery at room temperature (23°C), mice were housed at room temperature (23°C) or cold exposure (6°C) for 7 days with a chow diet (n=4 per group). (**C**) CCL22 concentration in dendritic cells from iWAT (n=8 per group). (**D** and **E**) Immunoblotting and quantification of CCL22 in dendritic cells from iWAT (n=3 per group). Data information: Results are presented as means ± SEM. In (**B** and **C**, **E**) $^{*}P \leq 0.05$ by nonpaired Student's t test.

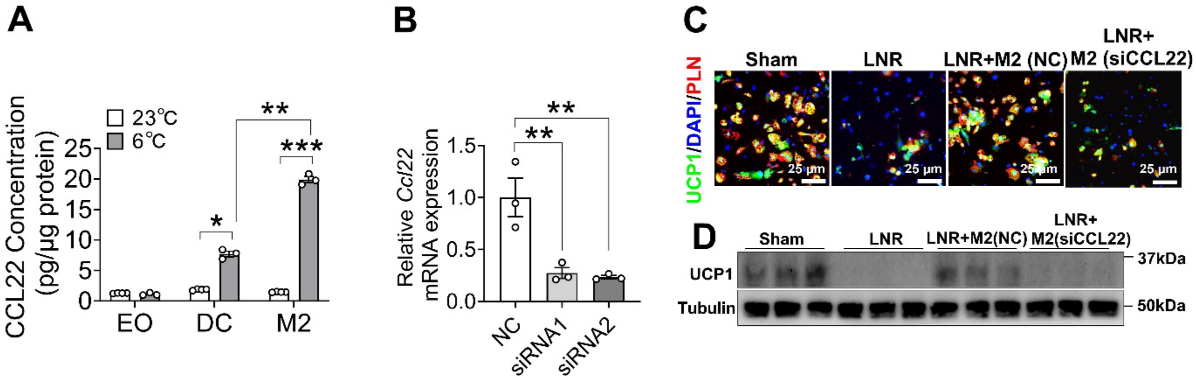


**Fig. S5 (related to Fig. 3): CCL22 is mainly secreted by M2 macrophages.**

(**A**) The CCL22 levels in eosinophils (EO), dendritic cells (DC), and M2 macrophages (M2) from iWAT. Ten-week-old male C57BL/6 mice were exposed at 23°C or 6°C for 7 days, and then EO, DC, and M2 macrophage cells were sorted from iWAT (n=3-4 per group). (**B**) mRNA expression of *Ccl22* in BMDM-derived M2 macrophages treated with negative control (NC) siRNA or siCCL22 (n=3 per group). (**C** and **D**) Immunofluorescence (**C**) and immunoblots (**D**) of UCP1 in beige adipocytes. Ten-week-old C57BL/6 male mice received LNR or sham in iWAT; then, mice were housed at a cold temperature (6°C) for 10 days with a chow diet. SVF cells were isolated from iWAT, and then co-cultured with BMDM-derived M2 macrophages with negative control siRNA treated or siCCL22 treated for 4 days, and beige adipocytes were induced for 5 days. Scale bar, 50 µm. Data information: Results are presented as means ± SEM. In (**A** and **B**) *P ≤ 0.05, **P ≤ 0.01, and ***P ≤ 0.001 by nonpaired Student’s t test.

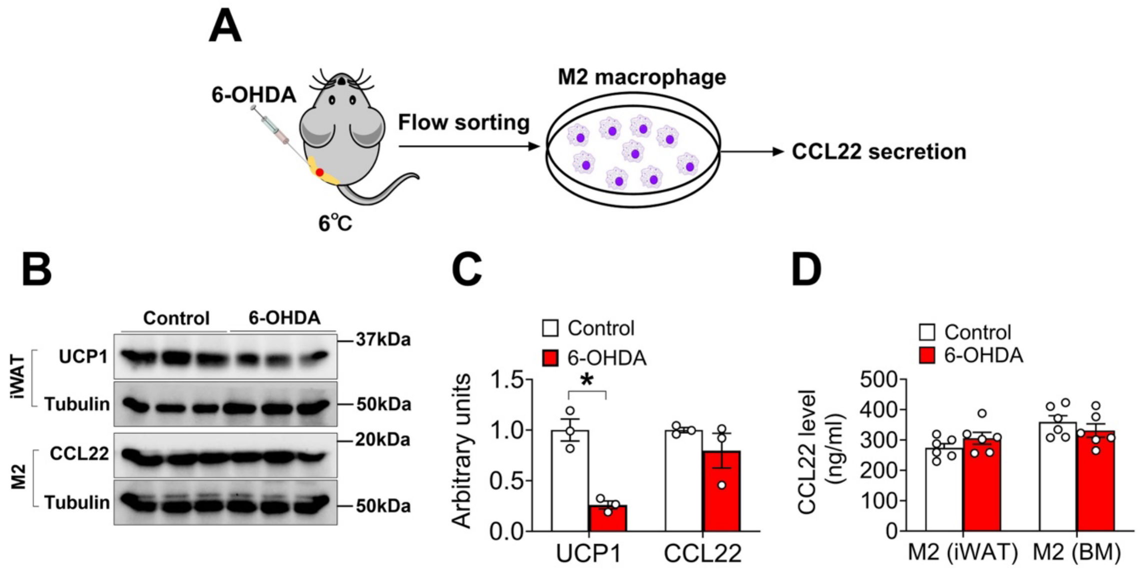


**Fig. S6 (related to Fig. 3): Macrophage-derived CCL22 is not regulated by the sympathetic system within iWAT.**

(**A**) Scheme of 6-OHDA treatment. Ten-week-old male C57BL/6 mice were subjected to bilateral sympathetic denervation of iWAT and lymph node by local injection of vehicle or 6-OHDA (10 mg/ml) directly into iWAT and lymph node. Afterward, the mice were cold (6°C) for 7 days and then sorted M2 macrophages from iWAT or isolated and induced M2 macrophages from bone marrow. M2 macrophages were cultured at 37°C for 4 days. (**B** and **C**) Immunoblots (**B**) and quantification (**C**) of UCP1 from iWAT or CCL22 in M2 macrophage derived from iWAT (n=3 per group). (**D**) CCL22 secretion by M2 macrophages derived from iWAT or bone marrow (n=6 per group). Data information: Results are presented as means ± SEM. In (**C** and **D**), *P ≤ 0.05 by nonpaired Student's t test.

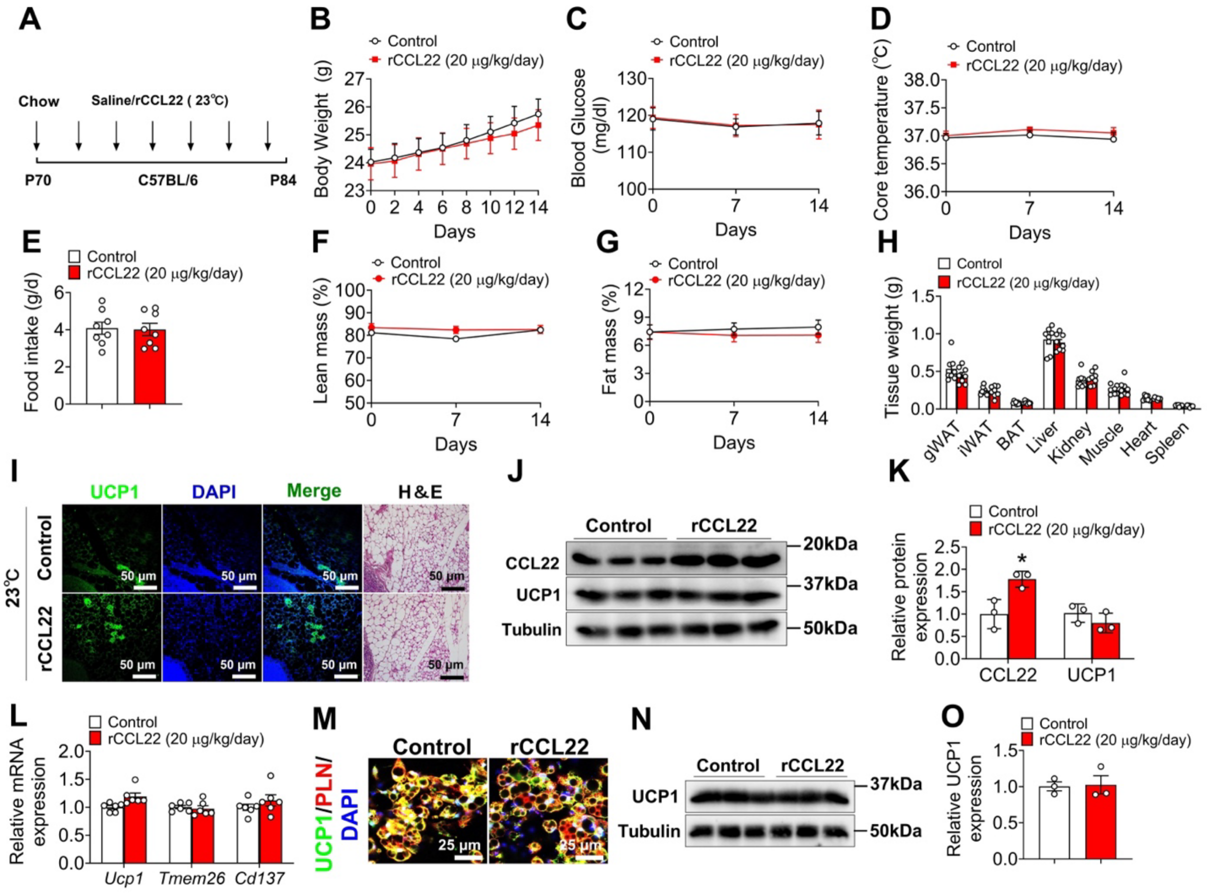


**Fig. S7 (related to Fig. 3): CCL22 does not promote beige adipocyte formation in the absence of cold challenge.**

(**A**) Schematic representation: 10-week-old C57BL/6 male mice received saline or rCCL22 (20 μg/kg/day) at 23 °C for 14 days with a chow diet. The mice were injected into bilateral iWAT with saline or rCCL22 every other day. (**B** to **H**) Body weight (**B**), blood glucose (**C**), core temperature (**D**), food intake (**E**), body composition (**F** to **G**), and tissue weight (**H**) (n=8 per group). (**I-L**) Immunofluorescence (**I**), immunoblots (**J**), and quantification (**K**) of UCP1, and mRNA expression (**L**) of *Ucp1, Cd137,* and *Tmem26* in iWAT (n=3-6 per group). Scale bar, 50 μm. (**M** to **O**) Immunofluorescence (**M**), immunoblots (**N**), and quantification (**O**) of UCP1 in beige adipocytes. SVF cells from 10-week-old male C57BL/6 mice were treated with vehicle or rCCL22 (10 ng/ml) for 4 days and then induced to differentiate into beige adipocytes for 5 days at 37°C. Scale bar, 25 μm.

Data information: Results are presented as means ± SEM. In (**B** to **D**, **F** to **G**), *P ≤ 0.05 by two-way ANOVA followed by post hoc Bonferroni tests. In (**E**, **H**, **K** and **L**, **O**) *P ≤ 0.05 by nonpaired Student’s t test.

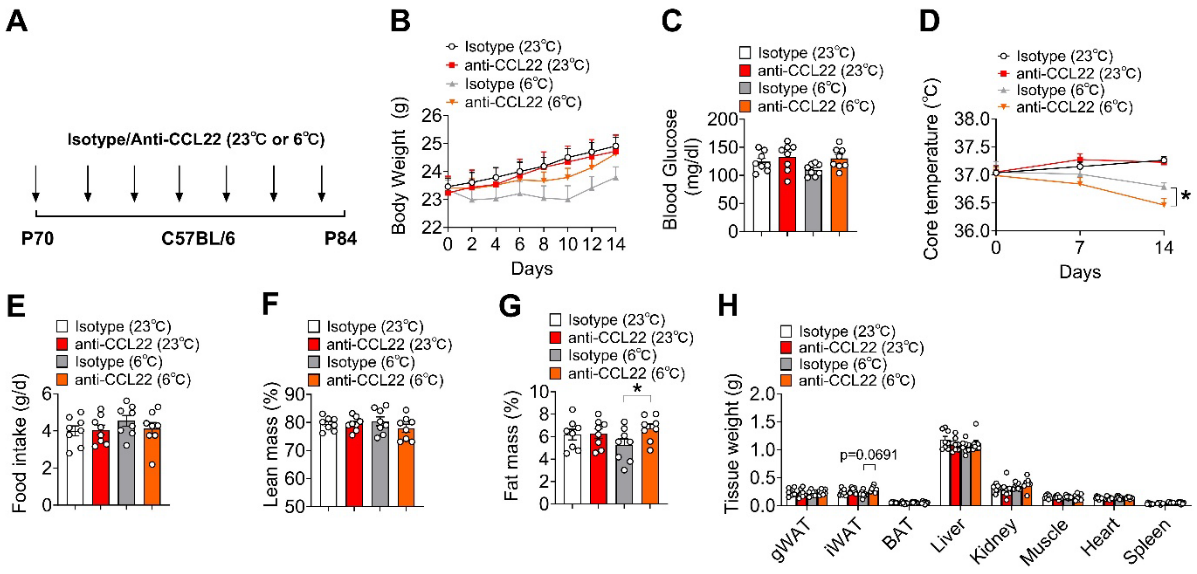


**Fig. S8 (related to Fig. 3): The metabolic effects of anti-CCL22 in WT mice.**

(**A**) Schematic representation: 10-week-old C57BL/6 male mice received isotype or CCL22 antibody (10 μg/kg/day) at 23°C or 6°C for 14 days with chow feeding. The mice were injected into bilateral iWAT with isotype or CCL22 antibodies every other day. (**B** to **H**) Body weight (**B**), blood glucose (**C**), core temperature (**D**), food intake (**E**), lean mass and fat mass (**F** to **G**), and tissue weight (**H**) (n=8 per group). Data information: Results are presented as means ± SEM. In (**B**, **D**), *P ≤ 0.05 by two-way ANOVA followed by post hoc Bonferroni tests. In (**C**, **E** to **H**) *P ≤ 0.05 by nonpaired Student's t test.

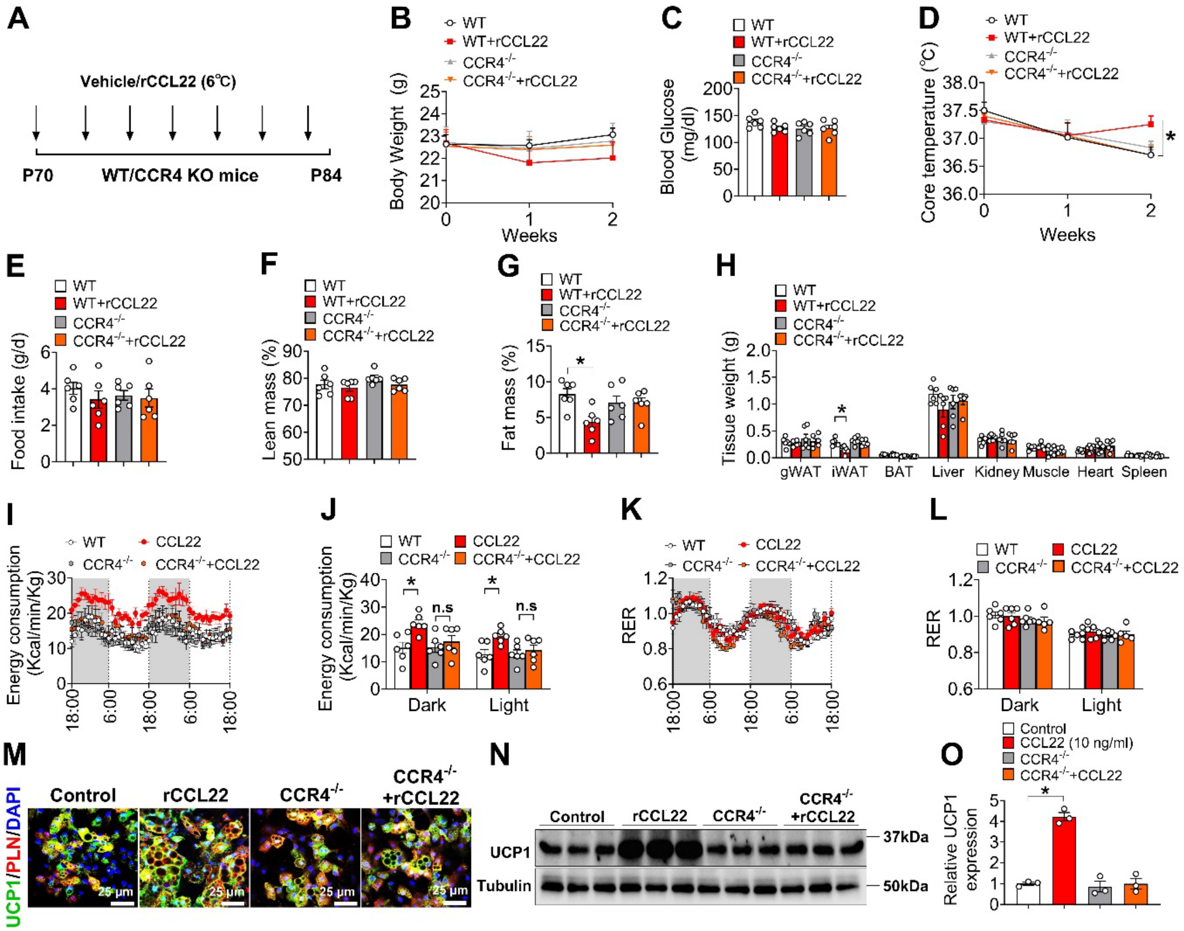


**Fig. S9 (related to Fig. 4): The metabolic effects of rCCL22 in WT and CCR4 knockout mice.** (**A**) Schematic representation: 8-week-old cas9 male mice were iWAT injected with AAV-sgRNAs-CCR4 [2 × $10^{13}$ genome copies (GCs)/ml] to generate a CCR4 deletion mouse model (CCR4$^{-/-}$). Littermate cas9 male mice receiving iWAT injections of the AAV-vehicle virus served as the WT control group. After a 2-week period (P70), both the WT and CCR4$^{-/-}$ mice were administered either vehicle or recombinant CCL22 (rCCL22) at a dosage of 20 μg/kg/day for 14 days. (**B** to **H)** Body weight (**B**), blood glucose (**C**), core temperature (**D**), food intake (E), lean mass and fat mass (F to G), and tissue weight (H) (n=8 per group). (**I** to **L**) Energy consumption rate (**I**) and quantification (**J**), RER (**K** and **L**). (**M** to **O**) Immunofluorescence (**M**), immunoblot (**N**), and quantification (**O**) of UCP1 from differentiated beige adipocytes (n=3 per group). SVF cells from 10-week-old male WT or CCR4$^{-/-}$ mice were treated with vehicle or rCCL22 (10 ng/ml) for 4 days and then induced to differentiate into beige adipocytes for 5 d at 31°C. Scale bar, 25 μm. Data information: Results are presented as means ± SEM. In (**B**, **D**, **I**, **K**), *P ≤ 0.05 by two-way ANOVA followed by post hoc Bonferroni tests. In (**C**, **E** to **H**, **J**, **L**, **O**) *P ≤ 0.05 by nonpaired Student's t test.

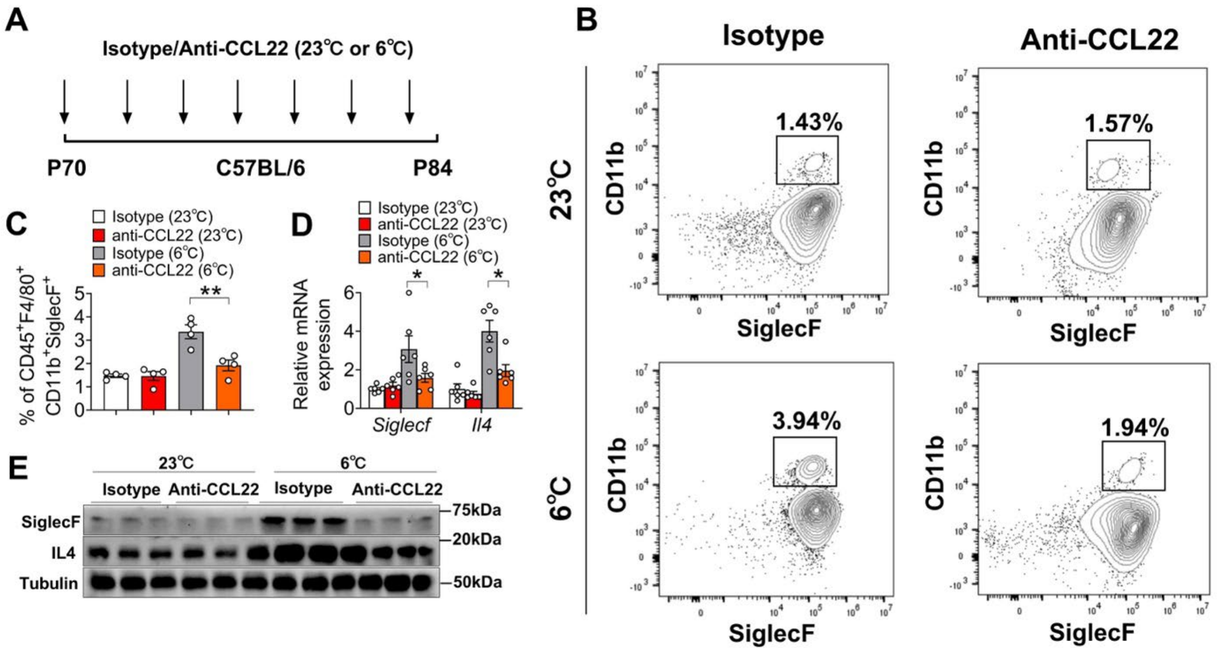


**Fig. S10 (related to Fig. 5): CCL22 is obligatory for cold-induced recruitment of eosinophils into iWAT.**

(**A**) Schematic representation: 10-week-old C57BL/6 male mice received isotype or CCL22 antibody (10 μg/kg/day) at 23°C or 6°C for 14 days with chow feeding. The mice were injected into bilateral iWAT with isotype or CCL22 antibodies every other day. (**B** and **C**) Flow cytometry analysis and quantification of eosinophils in iWAT (n=4 per group). (**D** and **E**) mRNA expression (**D**) and immunoblots (**E**) of the SiglecF and IL4 genes in iWAT (n=6 per group). Data information: Results are presented as means ± SEM. In (**C** and **D**) *P ≤ 0.05 by nonpaired Student's t test.

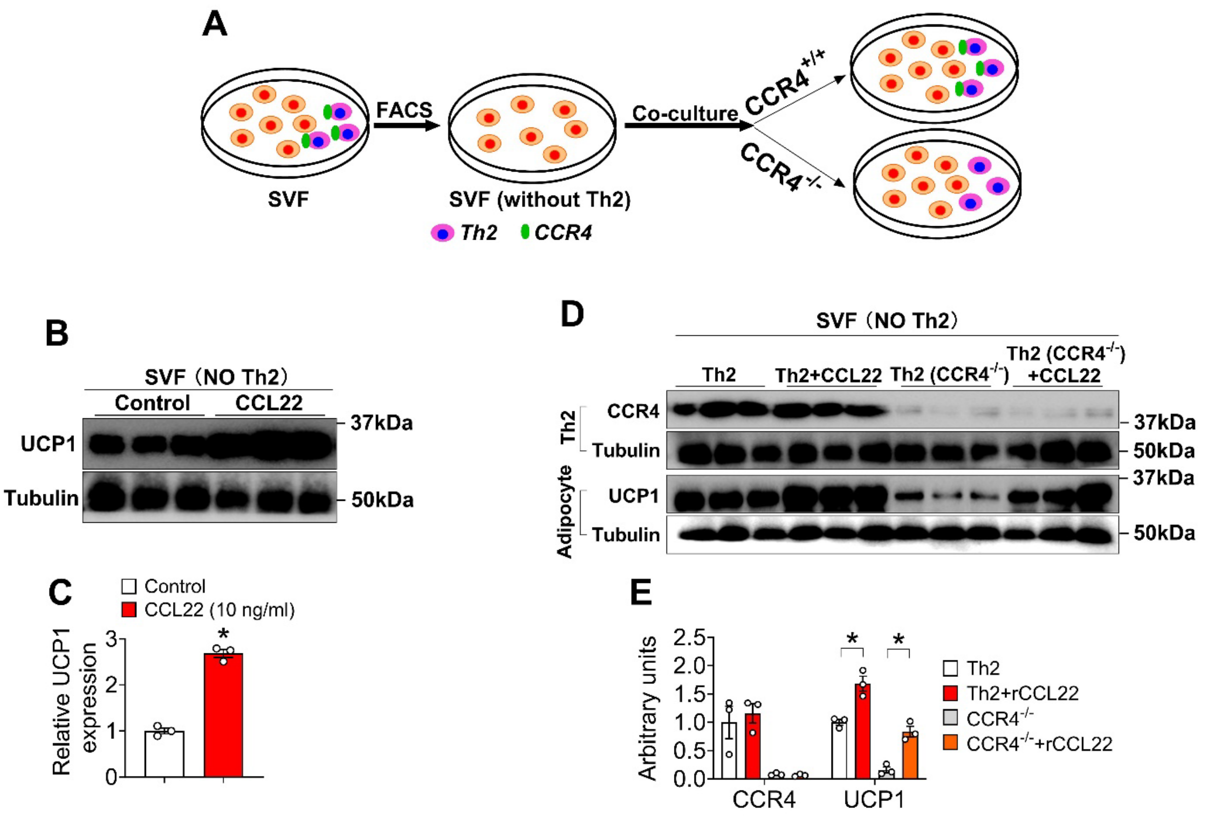


**Fig. S11 (related to Fig. 5): Th2 cells are not required for CCL22-induced beige adipocyte formation upon cold exposure.**

(**A**) Schematic representation picture. Th2 cells (from iWAT, transferred with vehicle or AAV-sgRNA-CCR4 virus for 3 days) and SVF (without Th2 cells) were cocultured for 2 days, treated with vehicle or rCCL22 for 4 days at 31°C and induced to form beige adipocytes for 5 days. SVF was obtained from 10-week-old cas9 male iWAT depots. Th2 cells were obtained from the bone marrow of 10-week-old cas9 male mice. (**B** and **C**) Immunoblot (**B**) and quantification (**C**) of UCP1 in beige adipocytes (n=3 per group). (**D** and **E**) Immunoblot (**D**) and quantification (**E**) of CCR4 in Th2 cells or UCP1 in beige adipocytes. Data information: Results are presented as means ± SEM. In (**C**, **E**), *$P \leq 0.05$ by nonpaired Student's t test.

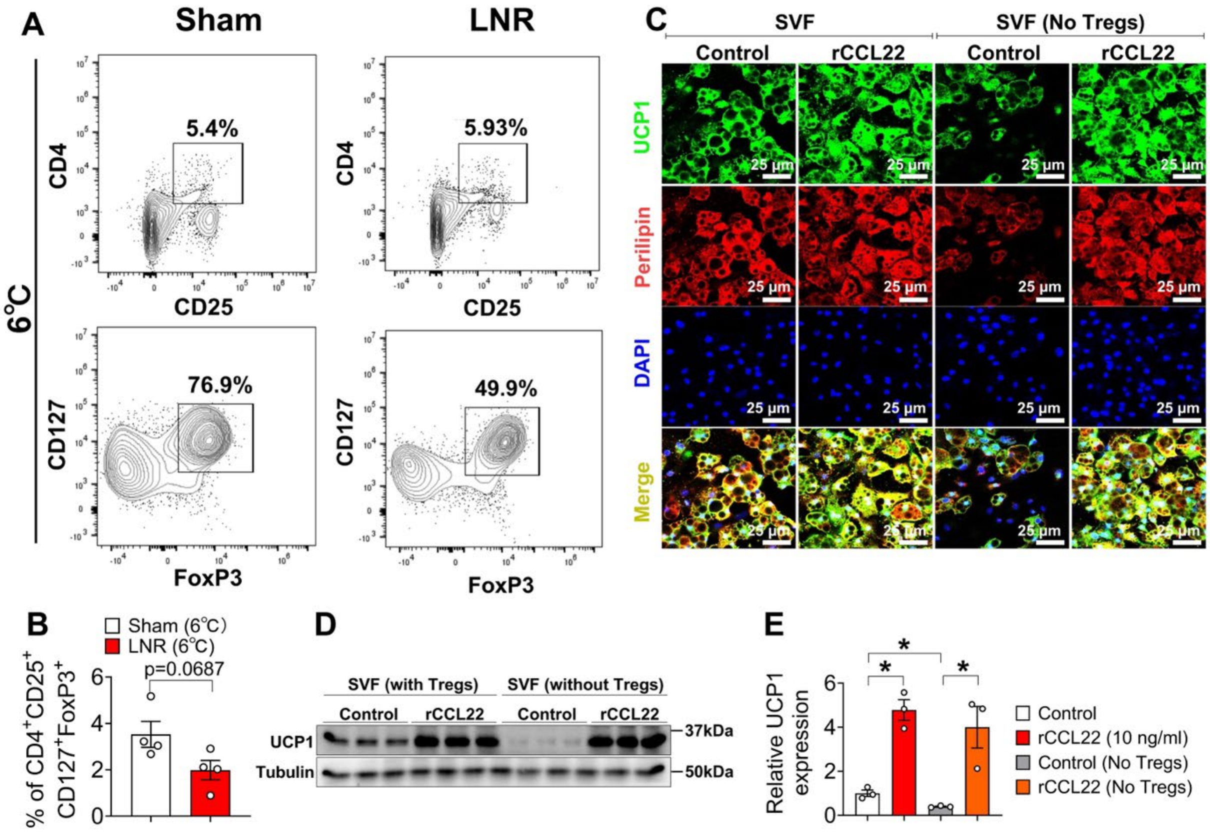


**Fig. S12 (related to Fig. 5): Treg cells are not involved in CCL22-induced beige adipocyte formation upon cold exposure.**

(**A** and **B**) Flow cytometry analysis and quantification of Treg cells ($CD4^+CD25^+CD127^+FoxP3^+$) in iWAT. Ten-week-old C57BL/6 male mice received LNR or sham in iWAT. After 10 days of recovery at room temperature (23°C), mice were stimulated by cold exposure (6°C) for 7 days with a chow diet (n=4 per group). (**C**) Immunofluorescence of UCP1 in beige adipocytes. SVF cells were extracted from 10-week-old C57BL/6 male mice and cultured at 31°C. SVF cells were treated with vehicle or rCCL22 for 4 days and then induced to differentiate into beige adipocytes for 5 days. Scale bar, 25 μm. (**D** and **E**) Immunoblotting and quantification of UCP1 in beige adipocytes (n=3 per group). Data information: Results are presented as means ± SEM. In (**B**, **E**), *P ≤ 0.05 by nonpaired Student's t test.

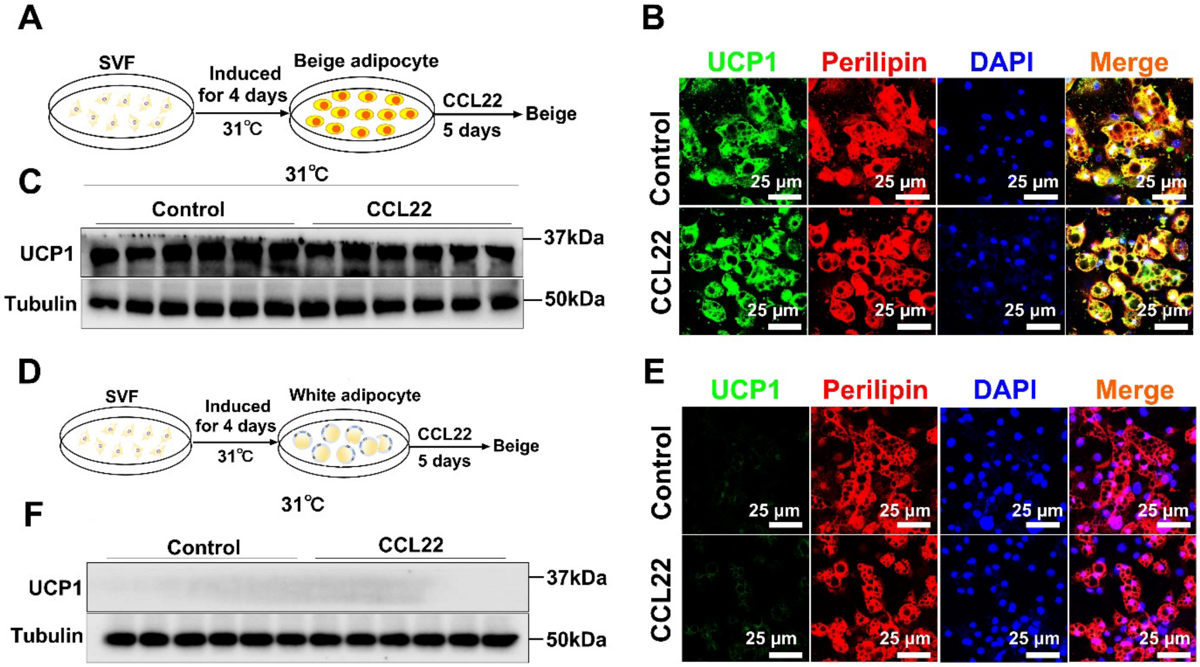


**Fig. S13 (related to Fig. 7): CCL22 does not exert its beiging effects on beige or white adipocytes.**

(**A**) Schematic representation: SVF cells from 10-week-old male mouse iWAT were induced to differentiate into beige adipocytes for 4 days and then treated with vehicle or rCCL22 (10 ng/ml) for 5 days at 31°C. (**B**) Immunofluorescence of UCP1 from differentiated treated beige adipocytes described (**A**). Scale bar, 25 μm. (**C**) Immunoblots of UCP1 from differentiated treated beige adipocytes described in (**A**) (n=3 per group). (**D**) Schematic representation: iWAT SVF cells from 10-week-old male mice were induced to differentiate into white adipocytes for 4 days and then treated with vehicle or rCCL22 (10 ng/ml) for 5 days at 31°C. (**E**) Immunofluorescence of UCP1 from vehicle or rCCL22 treated white adipocytes described in (**D**). Scale bar, 25 μm. (**F**) Immunoblots of UCP1 from vehicle or rCCL22 treated white adipocytes described in (**D**) (n=3 per group).

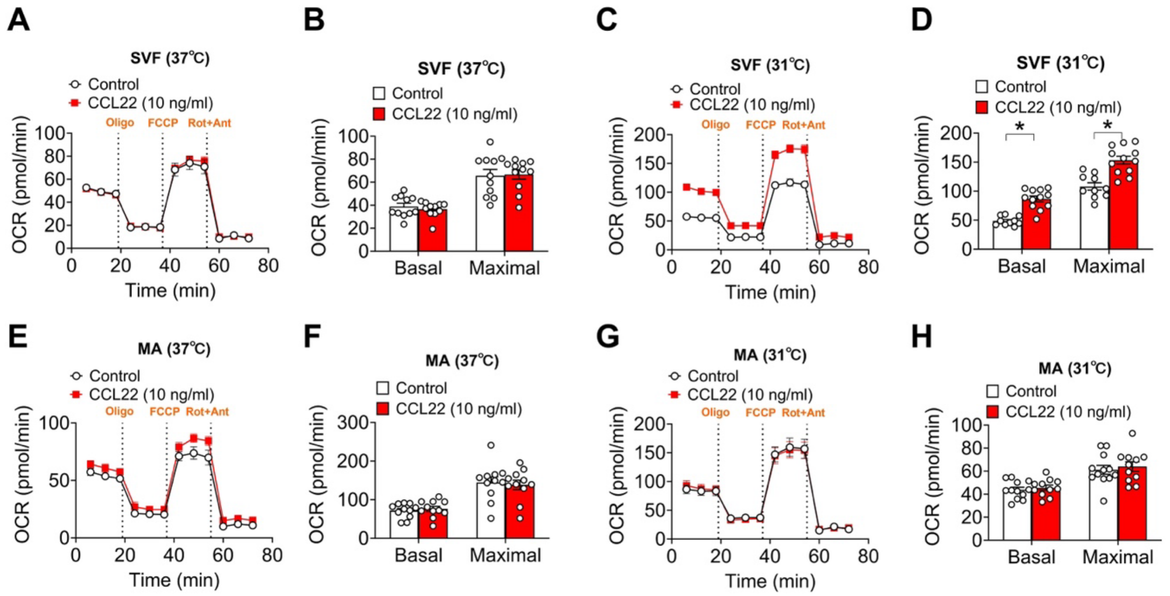


**Fig. S14 (related to Fig. 7): The effect of CCL22 on the oxygen consumption rate of SVF cells and beige adipocytes.**

(**A** to **D**) Oxygen consumption rate in beige adipocytes differentiated from 10-week-old male C57BL/6 mouse iWAT depots. SVF cells were treated with vehicle or rCCL22 (10 ng/ml) for 4 d at 31°C or 37°C. Then, beige adipocytes were induced for 5 days (n=12 per group). (**E** to **H**) Oxygen consumption rate in beige adipocytes differentiated from 10-week-old male C57BL/6 mouse iWAT depots. SVF cells were induced to differentiate into beige adipocytes for 4 days and then treated with vehicle or rCCL22 (10 ng/ml) for 4 d at 31°C or 37°C (n=12 per group). Data information: Results are presented as means ± SEM. In (**A**, **C**, **E**, **G**), *P ≤ 0.05 by two-way ANOVA followed by post hoc Bonferroni tests. In (**B**, **D**, **F**, **H**) *P ≤ 0.05 by nonpaired Student's t test.

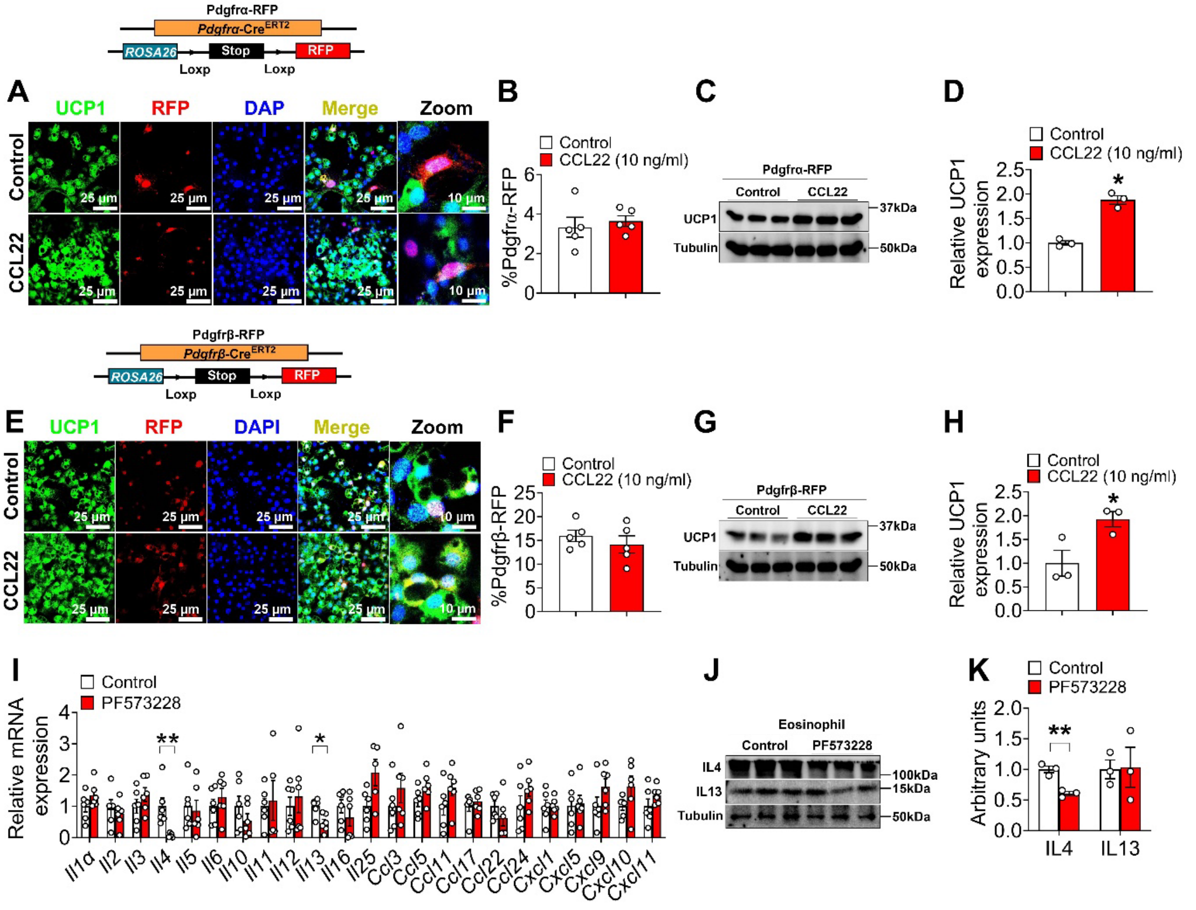


**Fig. S15 (related to Fig. 7): CCL22-induced beige adipocytes are largely not derived from Pdgfrα+ or Pdgfrβ+ lineage.**

(**A**) Immunofluorescence of UCP1 and RFP in Pdgfrα-RFP$^{+}$ derived beige adipocytes. SVF cells from the iWAT of 10-week-old male mice were treated with vehicle or rCCL22 (10 ng/ml) for 4 days, then induced to differentiate into beige adipocytes for 5 days at 31°C. Scale bar, 25 µm. (**B**) Quantification of the percentage of RFP+ cells that express endogenous UCP1 (n=5 per group). (**C** and **D**) Immunoblots (**C**) and quantification (**D**) of UCP1 protein from differentiated beige adipocytes described in (**A**). (**E**) Immunofluorescence of UCP1 and RFP in Pdgfrβ-RFP$^{+}$ derived beige adipocytes. SVF cells from the iWAT of 10-week-old male mice were treated with vehicle or rCCL22 (10 ng/ml) for 4 days, then induced to differentiate into beige adipocytes for 5 days at 31°C. Scale bar, 25 µm. (**F**) Quantification of the percentage of RFP+ cells that express endogenous UCP1 (n=5 per group). (**G** and **H**). Immunoblots (**G**) and quantification (**H**) of UCP1 protein from differentiated beige adipocytes described in (**E**). (**I**) mRNA expression of chemokines and cytokines in eosinophils. Eosinophils were sorted from iWAT of 10 10-week-old male mice and treated with vehicle or PF573228 (50 µM) for 2 days at 37°C. (**J** and **K**) Immunoblots (**J**) and

quantification (**K**) of IL4 and IL13 from eosinophils treated with vehicle or PF573228. Data information: Results are presented as means ± SEM. In (**B**, **D**, **F**, **H**, **I**, **K**) *$P \le 0.05$ and **$P \le 0.01$ by nonpaired Student's t test.

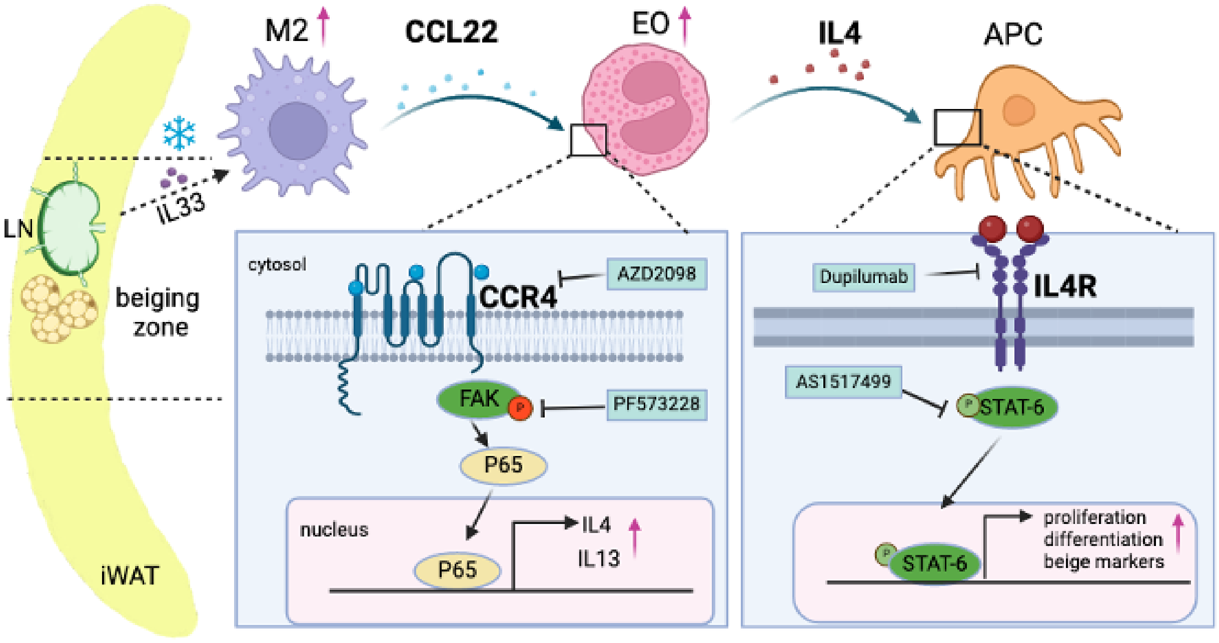


**Fig. S16 (related to Fig. 7): A schematic summary model of the CCL22-CCR4 axis in mediating LN-mediated APC beiging.**

Upon cold exposure, macrophage-derived chemokine CCL22 and its receptor CCR4 in eosinophils facilitate iWAT beiging around local LN (beiging zone). Specifically, the binding of CCL22 to CCR4 initiates FAK activation in eosinophils, which activates NF-kB, increasing IL-4 production. The secreted IL-4 activates STAT6 signaling in SMA+ APCs through IL-4R interaction. IL-33, released from LNs, may trigger this cascade.

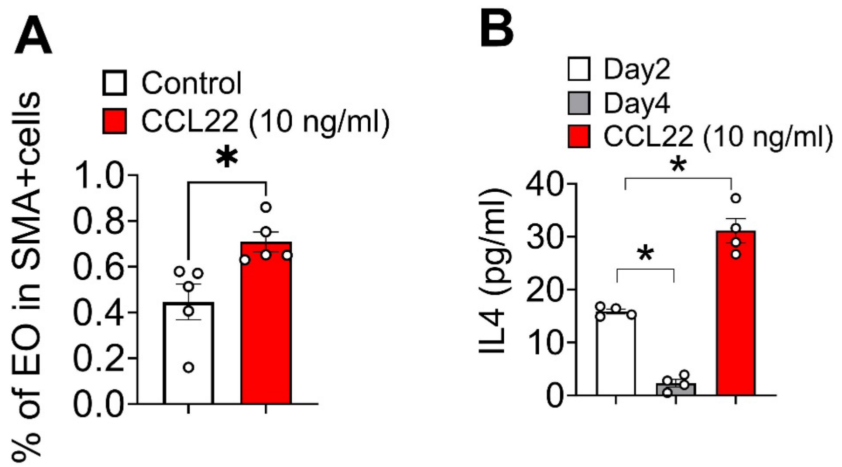


**Fig. S17 (related to Fig. 7): The effect of CCL22 on the number of eosinophils and IL4 levels.** (**A**) Flow analysis of the number of eosinophils in SMA-RFP$^+$ SVF cells. SVF cells from the iWAT of 10-week-old male mice were treated with vehicle or rCCL22 (10 ng/ml) for four days (n=5 per group). (**B**) The IL4 level in medium. SVF cells from the iWAT of 10-week-old male mice were treated with vehicle or rCCL22 (10 ng/ml) for four days (n=4 per group). Data information: Results are presented as means ± SEM. In (**A** and **B**) *P ≤ 0.05 by nonpaired Student's t test.

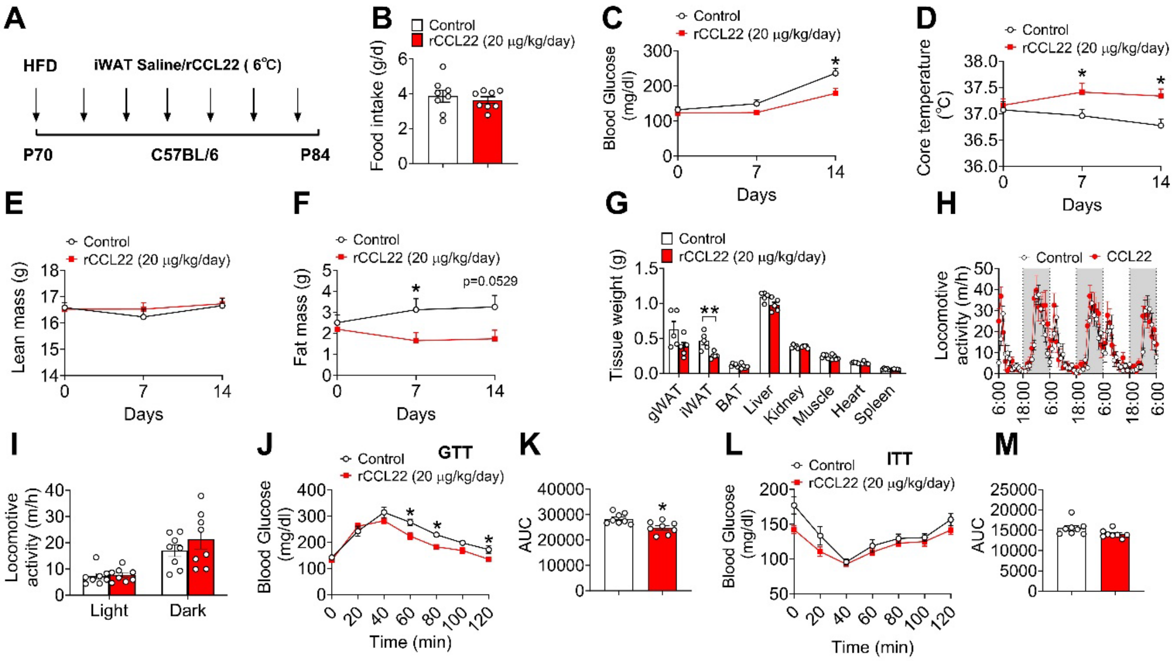


**Fig. S18 (related to Fig. 8): CCL22 treatment enhances metabolic effects in mice subjected to HFD and cold exposure.**

(**A**) Schematic representation: 10-week-old C57BL/6 male mice received saline or rCCL22 (20 μg/kg/day) at 6 °C for 14 days with a 60% high-fat diet (HFD). The mice were injected into bilateral iWAT with saline or rCCL22 every other day (n=8 per group). (**B** to **G**) Food intake (**B**), blood glucose (**C**), core temperature (**D**), body composition (**E** and **F**), and tissue weight (**G**) (n=8 per group). (**H** and **I**) Locomotive activity and quantification (n=8 per group). (**J** to **M**) Glucose tolerance test (GTT, 1 g/kg body weight) (**J** and **K**), insulin tolerance test (ITT, 1 U/kg body weight) (**L** and **M**) (n=8 per group). Data information: Results are presented as means ± SEM. In (**B**, **G**, **I**, **K**, **M**), *P ≤ 0.05 by nonpaired Student's t test. In (**C** to **F**, **H**, **J**, **L**), *P ≤ 0.05 by two-way ANOVA followed by post hoc Bonferroni tests.
